# Clinical and cost-effectiveness of the Lightning Process in addition to specialist medical care for paediatric chronic fatigue syndrome: randomised controlled trial

**DOI:** 10.1136/archdischild-2017-313375

**Published:** 2017-09-20

**Authors:** Esther M Crawley, Daisy M Gaunt, Kirsty Garfield, William Hollingworth, Jonathan A C Sterne, Lucy Beasant, Simon M Collin, Nicola Mills, Alan A Montgomery

**Affiliations:** 1 Centre for Child and Adolescent Health, Bristol Medical School: Population Health Sciences, University of Bristol, Bristol; 2 Bristol Medical School: Population Health Sciences, University of Bristol, Bristol; 3 Bristol Randomised Trials Collaboration, Bristol Medical School: Population Health Sciences, University of Bristol, Bristol; 4 Nottingham Clinical Trials Unit, School of Medicine, Queen’s Medical Centre, University of Nottingham, Nottingham, UK

**Keywords:** chronic fatigue syndrome, adolescent health, RCT

## Abstract

**Objective:**

Investigate the effectiveness and cost-effectiveness of the Lightning Process (LP) in addition to specialist medical care (SMC) compared with SMC alone, for children with chronic fatigue syndrome (CFS)/myalgic encephalitis (ME).

**Design:**

Pragmatic randomised controlled open trial. Participants were randomly assigned to SMC or SMC+LP. Randomisation was minimised by age and gender.

**Setting:**

Specialist paediatric CFS/ME service.

**Patients:**

12–18 year olds with mild/moderate CFS/ME.

**Main outcome measures:**

The primary outcome was the the 36-Item Short-Form Health Survey Physical Function Subscale (SF-36-PFS) at 6 months. Secondary outcomes included pain, anxiety, depression, school attendance and cost-effectiveness from a health service perspective at 3, 6 and 12 months.

**Results:**

We recruited 100 participants, of whom 51 were randomised to SMC+LP. Data from 81 participants were analysed at 6 months. Physical function (SF-36-PFS) was better in those allocated SMC+LP (adjusted difference in means 12.5(95% CI 4.5 to 20.5), p=0.003) and this improved further at 12 months (15.1 (5.8 to 24.4), p=0.002). At 6 months, fatigue and anxiety were reduced, and at 12 months, fatigue, anxiety, depression and school attendance had improved in the SMC+LP arm. Results were similar following multiple imputation. SMC+LP was probably more cost-effective in the multiple imputation dataset (difference in means in net monetary benefit at 12 months £1474(95% CI £111 to £2836), p=0.034) but not for complete cases.

**Conclusion:**

The LP is effective and is probably cost-effective when provided in addition to SMC for mild/moderately affected adolescents with CFS/ME.

**Trial registration number:**

ISRCTN81456207.

**What is already known on this topic?**

- Paediatric chronic fatigue syndrome (CFS)/myalgic encephalitis (ME) is relatively common with a negative impact on school, mood and quality of life.
- Even with effective treatment, a significant number of children have not recovered at 6 months.
- The Lightning Process (LP) is used by children with CFS/ME in the UK but with no evidence of effectiveness.

**What this study adds?**

- At 6 months, children who received LP in addition to SMC had better physical function, fatigue and less anxiety.
- At 12 months, children who received LP in addition to SMC had better fatigue, anxiety, depression and school attendance.
- Adding LP is probably cost-effective but not all children wish to take part.

## Introduction

Paediatric chronic fatigue syndrome (CFS) or myalgic encephalitis (ME) affects 0.57%–2.4%^1–4^of children and is disabling with important impacts on mood^5 6^ school attendance^4 7 8^ quality of life^9^ and family functioning.^10^ It is defined as generalised fatigue causing disruption of daily life, persisting after routine tests and investigations have failed to identify an obvious underlying cause.^11^ A minimum of 3 months of fatigue is required before the diagnosis can be made.^12^ On average, those affected miss a year of school overall and half are bedbound at some stage.^13 14^

There is a limited evidence base for treatment of paediatric CFS/ME.^12 15 16^ Three randomised trials have shown that cognitive–behavioural therapy (CBT) delivered individually,^17^ with biofeedback,^18^ or via the internet^19^ is effective at 6 months compared with waiting list or usual medical care. All three studies reported improvements in fatigue, school attendance and a reduction in disability. Family-focused CBT appears to be as effective as psychoeducation in terms of school attendance at 6 months and recovery at 24 months.^20^ ^21^ However, even with effective treatment, over a third of children^19 20^ have not recovered at 6 and 12 months^22^ and 21%^21^ to 36%^22^ are still unwell (eg, attending school <70% of the time) at 24 months. There is therefore an urgent need to find more effective treatments.

The Lightning Process (LP) is developed from osteopathy, life coaching and neurolinguistic programming and is used for a variety of conditions including CFS/ME. Clients read information, attend three group sessions and then receive follow-up phone calls.^23^ More than 250 children use LP for their CFS/ME each year in the UK (at a cost of ~£620 each), but there are no reported studies investigating its effectiveness, cost-effectiveness or side effects. LP is not available in the National Health Serivce (NHS). Having shown that recruitment, randomisation and data collection were feasible and acceptable,^24^ we conducted a randomised trial to investigate the effectiveness and cost-effectiveness of LP in addition to specialist medical care (SMC), compared with SMC alone, for children with CFS/ME.

## Methods

### Study design and participants

A detailed description of the study protocol has been reported.^25^ Between September 2010 and April 2013, we recruited participants after clinical assessment by the Bath/Bristol paediatric CFS/ME service, a large regional and national NHS specialist service. Children were diagnosed with CFS/ME after a thorough assessment which included screening for other disorders associated with fatigue.^12^ Baseline data were collected at this assessment. Children were eligible if they had CFS/ME, were aged 12–18, spoke English and were not housebound.

### Randomisation and masking

Allocation to trial arms was in equal proportions using minimisation by age (12–15/16–18 years) and gender, weighted towards minimising the imbalance in trial arms with probability 0.8. Allocation was concealed using a telephone-based interactive voice response system, created and maintained by the Bristol Randomised Trials Collaboration, and accessed by the recruiting researcher. This was an open study: the randomised intervention was conveyed during the recruitment interview so that participants, parents, therapists and researchers were aware of treatment allocation. Data analyses were conducted using masked treatment codes.

### Interventions

All participants were offered SMC^12^ which focused on improving sleep and using activity management to establish a baseline level of activity (school, exercise and social activity) which is then gradually increased. Sessions were delivered by a range of trained and supervised professionals including doctors, psychologists, physiotherapists and occupational therapists in family-based rehabilitation consultations. Follow-up sessions were either face to face or by telephone. The number and timing of the sessions were agreed with the family depending on each adolescent’s needs and goals. Those with significant anxiety or low mood were offered additional CBT. Participants could choose to use physiotherapist-delivered graded exercise therapy, which provides detailed advice about exercise and focuses on an exercise programme rather than other activities.

Participants randomised to SMC+LP were asked to read information about LP and complete an assessment form with their parents to identify their goals and describe what they had learnt. They then had a telephone call with an LP practitioner (online supplementary appendix 1) to discuss attending an LP course consisting of three 4-hour sessions on consecutive days run with groups of two to five young people. Each had a theory session with taught elements on the stress response, how the mind and body interact, and how thought processes can be either helpful or negative. This was followed by group discussion where the language used was discussed and in some cases challenged, and where participants were encouraged to think about what they could take responsibility for and change. In the practical session, participants identified a goal they wished to achieve (such as standing for longer) and were given different cognitive (thinking) strategies before and while the goal was attempted. They were also asked to identify a goal to attempt at home. After the course, young people were offered at least two follow-up phone calls with an LP practitioner.

10.1136/archdischild-2017-313375.supp1 Supplementary file 1

LP practitioners have completed a diploma through the Phil Parker Training Institute in Neurolinguistic Programming, Life Coaching and Clinical Hypnotherapy. This diploma is examined through written and practical examinations and is accredited by the British Institute of Hypnotherapy and NLP. Following the diploma, LP practitioners undertake a further course to learn the tools and delivery required for the LP after which they must pass both a practical and written examination. Practitioners undertake supervision and continuous professional development in order to further develop their skills and knowledge. They are regulated by the register of LP practitioners, adhere to a code of conduct and there is a Professional Conduct Committee that oversees complaints and professional practice issues.

### Outcomes

The primary outcome was the the 36-Item Short-Form Health Survey Physical Function Subscale (SF-36-PFS)^26^ analysed as a continuous variable collected at 6-month post-randomisation. Secondary outcomes were the SF-36-PFS at 3 and 12 months, and school attendance (days per week), the Chalder Fatigue Scale^27^ and quality-adjusted life years (QALYs, derived from the EQ-5D-Y)^28^ at 3, 6 and 12 months. Pain was measured by a Visual Analogue Scale (VAS) at 6 months. All were self-completed by participants. Participants also completed the Hospital Anxiety and Depression Scale (HADS)^29^ and the Spence Children’s Anxiety Scale (SCAS)^30^ at assessment, and at 3, 6 and 12 months. At 3, 6 and 12 months, parents completed an adapted four-item Work Productivity and Activity Impairment: General Health V2.0 questionnaire (V2.0)^31^ and a resource use questionnaire assessing their child’s health service use (eg, general practitioner or specialist care), educational service use (eg, school counsellor), health-related travel and other family costs.

Time windows for questionnaire return were prespecified as 6 weeks after the 3-month follow-up, 6 weeks before or up to 3 months after the 6-month follow-up and 3 months before or after the 12-month follow-up. Those who had not responded within 1 week were sent a reminder letter with a reduced set of questionnaires (SF-36-PFS, Chalder Fatigue Scale and school attendance). From February 2011, non-responders were telephoned by a researcher and the SF-36-PFS and Chalder Fatigue Scale were completed over the phone to improve follow-up rates.

### Sample size

We used a consensus definition for a small clinically important difference of 10 points on the SF-36-PFS.^32^ Thirty two to 50 participants in each arm are required to detect a between-group difference of 8 to 10 points on the SF-36-PFS (SD 10) at 6 months with 90% power and 1% two-sided significance. Allowing for 10% to 20% non-collection of primary outcome data, we aimed to recruit 80 to 112 participants.

### Statistical analysis

The statistical analysis plan was agreed by the study management group and published on our website prior to analyses. The primary analysis compared mean SF-36-PFS scores at 6 months according to randomised allocation among participants with measured outcomes, using multivariable linear regression adjusting for baseline values of the outcome, baseline age and gender. Similar regression analyses were conducted for secondary outcomes. Sensitivity analyses of the primary outcome adjusted for variables for which there was baseline imbalance; excluded those recruited up to 31 January 2011 preceding the protocol amendment; and used multiple imputation of missing data (see online supplementary appendix 1 for details). Missing items in partially completed scales (Chalder Fatigue and SF-36-PFS) or subscales (SCAS and HADS anxiety and depression) were imputed using the mean of completed items, if only one item (or two for the SCAS subscales) was missing. If more items were missing the whole scale or subscale was scored as missing. Twelve-month outcome data were analysed similarly. We conducted a repeated measures analysis using all follow-up SF-36-PFS scores, with and without an interaction between allocation arm and time, to investigate whether between-group differences remained constant over time. We estimated the Complier Average Causal Effect (CACE), using instrumental-variables linear regression estimated via the generalised method of moments, of LP among compliers, defined as participants in the SMC+LP arm who completed all of the LP course.

Prespecified subgroup analyses explored differences in treatment effect according to baseline age (<15 vs 15–17), gender, severity (none vs some school attendance at baseline) and comorbid anxiety (>or ≤12 on the HADS anxiety subscale) for the primary outcome, by adding an interaction term to the primary analysis multivariable linear regression model.

### Health economic analyses

We conducted a cost–utility analysis of SMC+LP from the health service and public sector perspective. We estimated the incremental net monetary benefit (iNMB) of SMC+LP versus SMC, at a threshold willingness to pay of £20 000 (~US$30 000) per QALY.^33^ In the primary analysis, we used the cost of LP charged to the trial (mean £567). In sensitivity analyses, we (1) used the price of LP outside of trial (£620; July 2014 price) and (2) estimated cost of providing the LP intervention within the UK health service (online supplementary table S1). SMC outpatient attendances were extracted from hospital records. Other healthcare use was based on parent report. Resource use was combined with 2013 unit costs including Agenda for Change pay bands effective from 1 April 2012 (online supplementary table S1).^34–37^ In the absence of a paediatric valuation for the EQ-5D-Y, we used the UK adult tariff.^38^ QALYs were estimated using the area under the curve.^39^ Incremental costs, QALYs and net benefits were adjusted for baseline values, age, gender and for variables where there was baseline imbalance. Non-parametric bootstrapping methods were used to calculate normally distributed 95% CIs around the iNMB. The probability that SMC+LP is cost-effective at varying willingness-to-pay thresholds was estimated using a cost-effectiveness acceptability curve. Where one item of the EQ-5D-Y was missing (n=3), the mean of the other domains (rounded to the nearest integer) replaced the missing value. A high proportion of participants had missing resource use data at 3, 6 and 12 months. Therefore, we conducted two analyses based on the complete case and multiply imputed datasets.

All analyses were conducted using Stata (Stata2013. Stata Statistical Software: Release 13.1).

## Results

**Figure 1:**
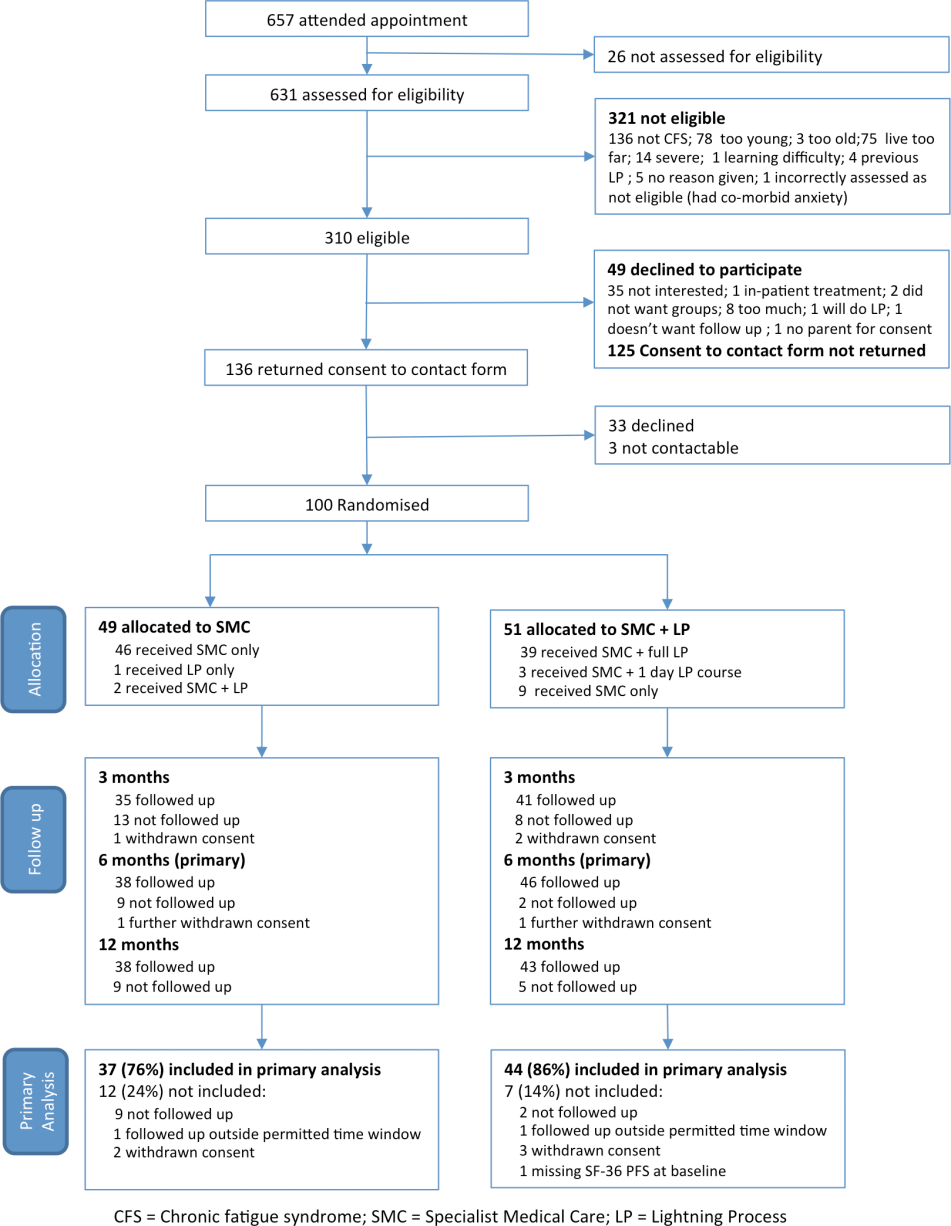
SMILE Consolidated Standards of Reporting**Trials** (CONSORT) trial profile. SF-36, the 36-Item Short-Form Health Survey.

Of 657 children assessed in the specialist CFS/ME clinic during the recruitment period, 631 were assessed for study eligibility and 310 were eligible (figure 1). Among those eligible, 136 consented to receiving further information and 100 were randomised: 49 to SMC only and 51 to SMC+LP. Recruitment was stopped after the 100th participant was randomised. Eligible children and adolescents who found out more about the trial but were not randomised had lower anxiety and depression scores and attended more school (online supplementary table S2). Participants’ mean age was 14 years, 76 were female and all described themselves as British. Participants were disabled by their fatigue: only seven were attending full-time school and 47 described themselves as attending 2 days or less school a week.

Participants’ characteristics at baseline were balanced between arms except for pain and anxiety (SCAS) scores (table 1), which was adjusted for in sensitivity analyses. Five participants withdrew from the study: two from the SMC and three from the SMC+LP arm. Outcome data were collected from 92 participants on at least one follow-up occasion. Baseline characteristics were similar between those who did (n=82) and did not (n=18) provide primary outcome data at 6 months (online supplementary table S3). The mean (SD) time between clinical assessment and primary outcome collection was 6.8 (1.0) and 6.8 (0.7) months in the SMC and SMC+LP arms, respectively. Treatment as allocated was received by 46 (94%) and 39 (76%) participants in the SMC and SMC+LP arms, respectively. Three participants in the SMC+LP arm received the LP course after completing the 6-month follow-up.

**Table 1:** Characteristics of the randomised participants at baseline

	SMC group		SMC plus LP group	
		N		N
Demographic characteristics				
Mean age (SD)	14.5 (1.6)	49	14.7 (1.4)	51
Number female (%)	38 (77.6)	49	38 (74.5)	51
Median months from onset of illness to baseline assessment (25th percentile, 75th percentile)	12 (7.0, 22.0)	49	12 (8.0, 18.0)	49
Clinical characteristics				
Mean SF-36 Physical Function score* (SD)	56.0 (21.5)	49	53.0 (18.8)	50
Mean Chalder Fatigue score† (SD)	25.1 (4.2)	49	25.0 (4.2)	50
Mean pain VAS† (SD)	42.4 (29.4)	48	51.6 (28.5)	48
Mean SCAS† (SD)	40.3 (20.1)	48	29.8 (16.9)	49
Mean HADS anxiety score† (SD)	10.4 (4.4)	48	8.8 (4.5)	51
Mean HADS depression score† (SD)	8.1 (4.4)	48	7.5 (3.1)	50
Mean EQ-5D score* (SD)	0.34 (0.36)	49	0.31 (0.34)	51
School attendance in the previous week, N (%)				
None	7 (14.3%)	49	6 (12.0%)	50
0.5 day	7 (14.3%)	49	5 (10.0%)	50
1 day	3 (6.1%)	49	3 (6.0%)	50
2 days	8 (16.3%)	49	8 (16.0%)	50
3 days	12 (24.5%)	49	12 (24.0%)	50
4 days	9 (18.4%)	49	12 (24.0%)	50
5 days	3 (6.1%)	49	4 (8.0%)	50

All results rounded to 1 d.p.

*Higher score=fewer symptoms, better function.

†Higher score=more symptoms, poorer function.

HADS, Hospital Anxiety and Depression Scale; SCAS, Spence Children’s Anxiety Scale; SF-36, the 36-Item Short-Form Health Survey; VAS, Visual Analogue Scale.

**Figure 2:**
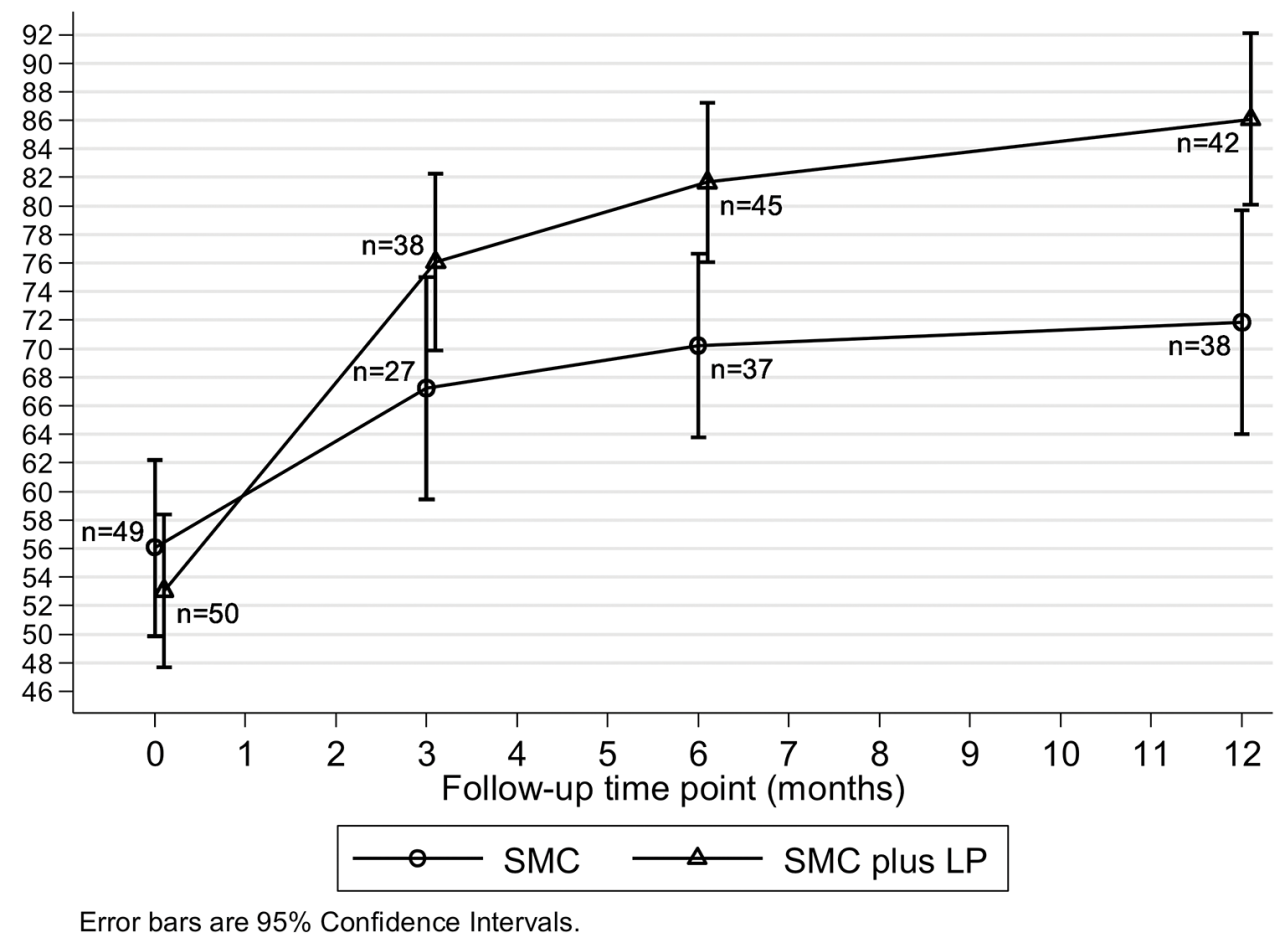
Mean SF36 physical function over time. LP, Lightning Process; SF-36, the 36-Item Short-Form Health Survey; SMC, specialist medical care.

Mean SF-36 physical function improved more over time in participants allocated to SMC+LP than in those allocated to SMC (figure 2). Participants allocated to SMC+LP had better physical function at 6 months than those allocated to SMC (table 2, adjusted difference in means 12.5 (95% CI 4.5 to 20.5), p=0.003). This difference increased to 15.1 (95% CI 5.8 to 24.4, p=0.002) at 12 months. These differences were similar when additionally adjusted for baseline anxiety (SCAS) and pain (VAS), when analyses were restricted to participants recruited from February 2011, and with multiple imputation of missing data (table 2). The average between-arm difference in physical function across both 6 and 12-month follow-up was 14.4 (95% CI 7.3 to 21.5), p<0.001. The estimated effect of LP (using CACE analyses) among compliers at 6 and 12 months was increased compared with the intention-to-treat (ITT) estimate (table 2). There was little evidence that the effect of LP+SMC compared with SMC on the primary outcome differed according to baseline age, anxiety or school attendance (all interaction p values>0.3). There was weak evidence (online supplementary table S4) that the effect in males (adjusted difference in means 26.6 (95%CI 8.9 to 44.3)) was greater than that in females (adjusted difference in means 9.0 (95% CI 0.2 to 17.8)) with an interaction p value of 0.08.

**Table 2:** Primary outcome

SF-36 physical function	SMC group		SMC plus LP group		Crude difference in means (95% CI), p value	Adjusted difference in means* (95% CI), p value	N	Adjusted difference in means† (95% CI), p value	N
	Mean	N	Mean	N					
Baseline	56.0	49	53.0	50					
6 months (primary outcome)‡	70.2	37	81.7	45	11.5 (3.1 to 19.8), 0.008	12.5 (4.5 to 20.5), 0.003	81	12.9 (3.6 to 22.1), 0.007	76
Children recruited from 1 February 2011	70.5	34	81.4	39	10.9 (1.8 to 20.0), 0.020	11.8 (3.2 to 20.3), 0.008	72	13.1 (3.3 to 22.8), 0.009	68
With imputation of missing data	70.9	49	81.1	51	10.2 (2.2 to 18.2), 0.013	11.3 (3.8 to 18.9), 0.004	100	11.8 (3.6 to 19.9), 0.005	100
Effect among compliers (CACE)					15.2 (5.0 to 25.3), 0.003	16.6 (6.9 to 26.2), 0.001	81	17.5 (7.1 to 28.0), 0.001	76
12 months‡	71.8	38	86.1	42	14.2 (4.6 to 23.8), 0.004	15.1 (5.8 to 24.4), 0.002	79	16.4 (6.1 to 26.8), 0.002	73
With imputation of missing data	73.1	49	85.5	51	12.4 (3.3 to 21.5), 0.008	12.6 (4.0 to 21.3), 0.005	100	14.7 (5.6 to 23.9), 0.002	100
Effect among compliers (CACE)					16.2 (5.6 to 26.7), 0.003	17.1 (7.0 to 27.3), 0.001	79	18.6 (6.9 to 30.4), 0.002	73
Average of 3, 6 and 12 month differences§						13.6 (6.7 to 20.4),<0.001	90	13.5 (6.0 to 21.0),<0.001	84
Average of 6 and 12 month differences§						14.4 (7.3 to 21.5),<0.001	87	14.9 (7.0 to 22.7),<0.001	81

*Adjusted for age, gender and baseline outcome.

†Adjusted for age, gender, baseline outcome, baseline Spence Children’s Anxiety Scale and Visual Analogue Scale.

‡Higher score=fewer symptoms, better function.

§Based on a repeated measures analysis that was additionally adjusted for time point as a categorical variable.

CACE, Complier Average Causal Effect; LP, Lightning Process; SF-36, the 36-Item Short-Form Health Survey; SMC, specialist medical care.

Participants in the SMC+LP arm had less fatigue (adjusted difference in means −4.7 (95% CI −7.9 to −1.6), p=0.003) (table 3) than those allocated to SMC and a greater improvement in anxiety symptoms measured by both the HADS (−3.3, (95% CI −5.6 to −1.0), p=0.005) and the SCAS (−8.7, (95% CI −16.9 to −0.5), p=0.039) scores at 6 months. The difference in means in fatigue score and HADS anxiety score were somewhat smaller at 12 months (−3.2 (95% CI −6.3 to −0.1) and −2·8 (−4.7 to –0.8) respectively). However, the difference in means in SCAS anxiety was greater at 12 months (−12.1 (95% CI −20.1 to –4.1) and there was evidence that there was less depression among participants allocated to SMC+LP than those allocated to SMC at 12 months (adjusted difference in means in HADS depression score −1.7 (95% CI −3.3 to −0.2) p=0.030). Participants allocated to SMC+LP had better school attendance at 12 months than those allocated to SMC (adjusted difference in means 0.9 days of school per week (95% CI 0.2 to 1.6) p=0.018). Pain scores were lower in participants allocated to SMC+LP compared with those allocated to SMC at both 6 and 12 months, but CIs were wide.

**Table 3:** Secondary outcomes

	SMC group		SMC plus LP group		Crude difference in means (95% CI), p value	Adjusted difference in means* (95% CI), p value	N	Adjusted difference in means† (95% CI), p value	N
	Mean	N	Mean	N					
Chalder Fatigue score 6 months‡	19.8	37	14.4	44	−5.4 (−8.6 to 2.1), 0.001	−4.7 (−7.9 to 1.6), 0.003	80	−5.4 (−8.9 to 1.9), 0.003	76
Chalder Fatigue score 12 months‡	15.7	38	12.3	42	−3.4 (−6.6 to 0.1), 0.041	−3.2 (−6.3 to 0.1), 0.045	79	−4.0 (−7.2 to 0.7), 0.017	74
Pain VAS 6 months‡	32.8	28	23.4	33	−9.5 (−23.5 to 4.6), 0.183	−11.3 (−23.0 to 0.3), 0.057	58	−9.3 (−21.1 to 2.6), 0.124	58
Pain VAS 12 months‡	32.0	27	21.8	32	−10.2 (−24.6 to 4.2), 0.161	−9.4 (−21.5 to 2.7), 0.125	56	−6.5 (−19.4 to 6.5), 0.321	54
SCAS 6 months‡	37.4	28	24.7	33	−12.7 (−22.0 to 3.3), 0.009	−8.7 (−16.9 to 0.5), 0.039	61	−10.0 (−18.5 to 1.5), 0.022	58
SCAS 12 months‡	36.3	27	19.6	31	−16.7 (−25.9 to 7·5), 0.001	−12.1 (−20.1 to 4.1), 0.004	56	−14.5 (−22.4 to 6.7),<0.001	52
HADS anxiety score 6 months‡	9.7	28	6.1	33	−3.7 (−6.0 to 1·3), 0.003	−3.3 (−5.6 to 1.0), 0.005	60	−3.5 (−5.6 to 1.5), 0.001	57
HADS anxiety score 12 months‡	8.3	27	5.3	33	−3.1 (−5.2 to 0.9), 0.006	−2.8 (−4.7 to 0.8), 0.006	59	−2.6 (−4.7 to 0.4), 0.019	53
HADS depression score 6 months‡	5.9	28	4.2	33	−1.7 (−4.0 to 0.6), 0.141	−1.6 (−3.9 to 0.7), 0.161	59	−1.5 (−3.5 to 0.5), 0.129	57
HADS depression score 12 months‡	4.6	27	2.8	33	−1.9 (−3.6 to 0.2), 0.033	−1.7 (−3.3 to 0.2), 0.030	58	−1.8 (−3.4 to 0.1), 0.037	53
School/college attendance in the previous week 6 months§ (days)	2.6	37	3.2	41	0.7 (−0.1 to 1.4), 0.083	0.7 (0.0 to 1.4), 0.064	77	0.6 (−0.2 to 1.4), 0.135	72
School/college attendance in the previous week 12 months§ (days)	3.1	36	4.1	34	1.0 (0.2 to 1.7), 0.010	0.9 (0.2 to 1.6), 0.018	69	1.0 (0.2 to 1.8), 0·012	65

*Adjusted for age, gender and baseline outcome.

†Higher score=more symptoms, poorer function.

‡Adjusted for age, gender, baseline outcome, baseline SCAS and VAS (as appropriate).

§Higher score=fewer symptoms, better function.

HADS, Hospital Anxiety and Depression Scale; LP, Lightning Process; SCAS, Spence Children’s Anxiety Scale; SF-36: The 36-Item Short-Form Health survey; SMC, specialist medical care; VAS, Visual Analogue Scale.

Five adverse events were reported (three in the SMC+LP arm). Four were related to participants and one to a parent. None were attributed to either SMC or LP. Physical function at 6 months deteriorated in nine participants, of whom eight were in the SMC arm. Five of the nine had deterioration of ≤10 on the SF-36 physical function subscale (range 0–100) which is less than the minimal clinically important difference.

**Figure 3:**
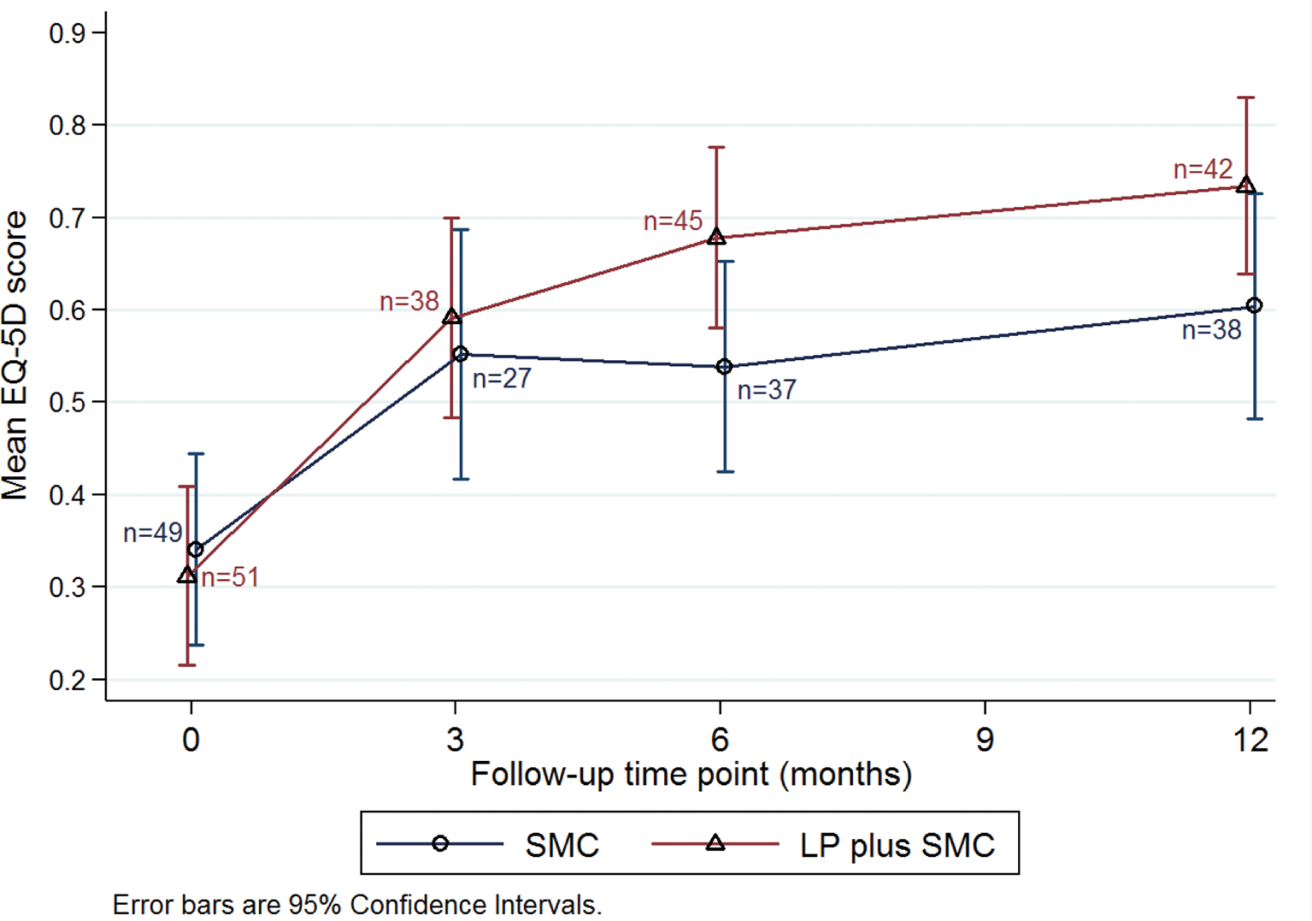
Mean EQ-5D scores, by treatment group. LP, Lightning Process; SMC, specialist medical care.

EQ-5D-Y questionnaires were completed by 65, 82 and 80 participants at 3, 6 and 12 months, respectively (figure 3); 56 completed EQ-5D-Y at all three follow-up time points. EQ-5D-Y scores were generally higher in the SMC+LP group. Differences in QALYs were evident at 12 months in the multiple imputation dataset (table 4, adjusted difference in means 0.095 QALYs (95% CI 0.030 to 0.160), p=0.004), but in the complete case dataset the CI included zero (adjusted difference in means 0.080 QALYs (95% CI −0.064 to 0.225), p=0.276).

**Table 4:** MI and complete case analysis of total HC+LP costs and QALYs and NMB (£20 k) at 6 and 12 months; by treatment group, all adjusted for baseline value, age, sex, baseline SCAS and baseline VAS

	SMC			SMC plus LP			Incremental difference	
	Mean	(SE)	n	Mean	(SE)	n	(95% CI)	n
6-month complete case								
Total cost (£)	942	(89)	13	1563	(127)	21	621 (323 to 919)	34
QALYs	0.252	(0.021)	22	0.259	(0.016)	32	0.008 (−0.057 to 0.073)	34
NMB at £20 000 per QALY	4225	(578)	13	3762	(461)	21	−464 (−1852 to 925)	34
6-month imputed								
Total cost (£)	1123	(66)	49	1517	(54)	51	394 (236 to 553)	100
QALYs	0.247	(0.015)	49	0.274	(0.014)	51	0.026 (−0.015 to 0.068)	100
NMB at £20 000 per QALY	3819	(328)	49	3954	(276)	51	135 (−733 to 1003)	100
12-month complete case								
Total cost (£)	1369	(160)	11	1814	(211)	16	445 (−57 to 947)	27
QALYs	0.551	(0.039)	21	0.597	(0.032)	30	0.080 (−0.064 to 0.225)	27
NMB at £20 000 per QALY	9454	(1202)	11	10 615	(1113)	16	1161 (−1966 to 4289)	27
12-month imputed								
Total cost (£)	1612	(84)	49	2002	(67)	51	390 (189 to 591)	100
QALYs	0.533	(0.025)	49	0.628	(0.021)	51	0.095 (0.030 to 0.160)	100
NMB at £20 000 per QALY	9042	(521)	49	10 551	(427)	51	1508 (148 to 2869)	100

HC, health care; LP, Lightning Process; MI, multiple imputation; NMB, net monetary benefit; QALY, quality-adjusted life years; SCAS, Spence Children’s Anxiety Scale; SMC, specialist medical care; VAS, Visual Analogue Scale.

Complete healthcare use questionnaires were returned by between 55 (55% at 12 months) and 56 (56% at 3 and 6 months) participants, but only 30 (30%) completed these questionnaires at all three time points (see online supplementary table S5 for details). The initial cost of LP was not fully offset by marginally lower costs of other care over the 12-month period. The incremental cost (table 4) of SMC+LP was higher in both complete case (difference in means £445, (95% CI −57 to 947), p=0.082) and multiple imputation datasets (difference in means £390, (95% CI 189 to 591), p=0.000).

**Figure 4:**
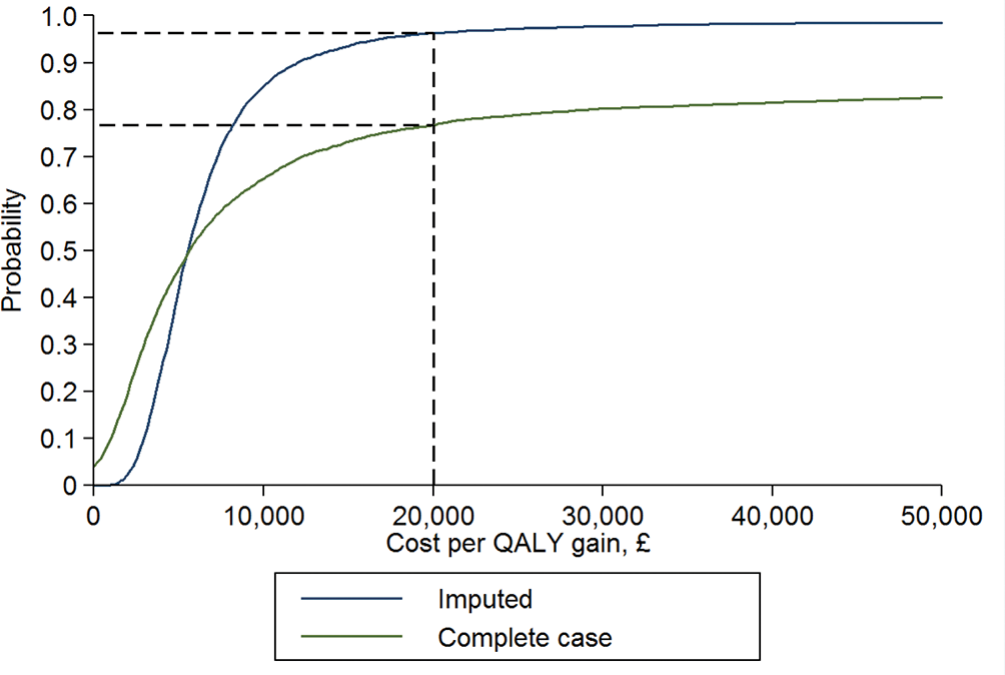
Cost-effectiveness acceptability curves* based on complete case and multiply imputed estimates of incremental costs and QALYs of SMC plus LP at 12 months. *For any selected willingness to pay for a QALY (x axis), the probability that SMC plus LP is cost-effective can be estimated by reading up from that point on the x axis to the curve and then reading across to the probability (y axis). The dashed lines represent the lower National Institute for Health and Care Excellence threshold of £20 000 per QALY gained. QALY, quality-adjusted life years; LP, Lightning Process; SMC, specialist medical care.

Table 4 shows that in the multiple imputation dataset there was good evidence that SMC+LP was more cost-effective than SMC alone (iNMB £1508 (95% CI £148 to £2869), p=0.034), although the evidence was much weaker in the complete case dataset (figure 4). Sensitivity analyses varying the unit cost of LP treatment made no difference to this conclusion (online  supplementary table S6, supplementary figure S1). Sensitivity analyses assuming costs and QALYs are not missing at random^40^ did not alter the conclusion that SMC+LP was likely to be cost-effective, but reduced the strength of the evidence.

10.1136/archdischild-2017-313375.supp2 Supplementary file 2

## Discussion

This is the first randomised trial investigating the effectiveness of the LP for any condition. It is the first trial that has demonstrated the effectiveness of an intervention other than CBT for paediatric CFS/ME. The addition of the LP to SMC improved physical function at 6 months in adolescents with CFS/ME and this difference increased at 12 months. The addition of LP also improved fatigue and anxiety at 6 months, and fatigue, anxiety and depression at 12 months. Participants in the LP arm were attending 1 day more of school a week at 12 months. The initial cost of LP was not fully offset by lower subsequent costs of healthcare, but the improvements in health-related quality of life meant that SMC+LP is probably cost-effective using a threshold for a QALY of £20 000 (~US$30 000). Participants in the Specialist Medical Intervention and Lightning Evaluation (SMILE) trial did not have any serious adverse events attributable to either treatment arm. The majority of those who experienced a deterioration in physical function had a deterioration of ≤10 on the SF-36 PFS. The lack of serious adverse events is consistent with other treatment trials in CFS/ME.^40^

Strengths of the study include its randomised design and that we followed patients up for 12 months. Participants received SMC that is currently being delivered in the UK Health Service by a multidisciplinary team, and the LP as it is currently provided. More participants were lost to follow-up in the SMC arm, but baseline characteristics were similar in those followed and not followed up. Complete healthcare use questionnaires were returned by only 55 or 56 participants at each time point. We used multiple imputation to correct for potential bias due to missing data and conducted sensitivity analyses restricted to participants recruited after the protocol changed to collect primary outcome data by telephone, which improved follow-up rates suggesting that results were robust. We predefined the clinically important difference (10 points) on the SF-36-PFS and the difference in means was greater than this at both 6 and 12 months. The study was not blinded, so that patient-reported outcomes may have been affected by participants’ knowledge of the group to which they were randomised. Only 36 (70%) of those allocated LP attended the full course prior to the 6-month follow-up but we estimated the effect in all those who completed the full LP course.

The LP may not be suitable for all children and adolescents. Fewer than 30% of eligible children were randomised. We do not know why the majority did not want to take part in the trial but it may be because they did not want to take part in groups or travel for three consecutive days. We felt that it would be unethical to have a control group without treatment, and therefore we only know that LP is effective in addition to SMC and not whether it is effective on its own. We only recruited children aged 12 and over who were not housebound and who spoke English. We do not know whether LP is effective, acceptable or feasible for those who are severely affected, less than 12 years old or do not speak English.

Participants in both treatment arms improved. Those receiving SMC alone had a mean improvement that was similar to that seen in adults receiving CBT or Graded Exercise Therapy (GET).^40^ The improvement in SF-36-PFS in those receiving SMC+LP is consistent with those receiving treatment in previous paediatric trials investigating both family based and individual CBT.^17 20^ The participants in our study who received SMC only did not improve as much as other trials investigating CBT^17 20^ which may be because on average they had less than half the number of treatment sessions. As we did not compare LP with either a full course of only CBT or GET, we do not know if LP is more of less effective than either of these treatment approaches.

Participants in the SMC+LP arm maintained or increased improvements compared with SMC alone at 12 months and this was true for both the ITT and the CACE analyses. This is in contrast to previous trials investigating internet-based CBT where the treatment effects were sustained but the difference between the two trial arms was reduced at 12 months compared with 3 months^19 22^ and family-focused CBT versus psychoeducation where treatment differences at 3 months were not maintained at 6 or 12 months.^20^

There is only one study^23^ investigating LP which used qualitative interviews to explore the views of nine 14–26 year olds about their experiences. The main difference between LP and CBT appears to be the emphasis placed on physiological responses and causal attributions^23^ but we do not know whether these explain the greater effectiveness of LP. We do not know which aspects of the LP are the most important or helpful. Some young people who received LP value the theory, others the practical sessions or the homework.^23^ Further research is needed to understand why LP improves outcomes at 6 and 12 months and which aspects of the LP contribute to its effectiveness.
