# Notice of correction and clarification: *Clinical and cost-effectiveness of the Lightning Process in addition to specialist medical care for paediatric chronic fatigue syndrome: randomised controlled trial*

**DOI:** 10.1136/archdischild-2017-313375corr1

**Published:** 2019-07-11

## Notice of correction and clarification

This article (*Archives of Disease in Childhood* 2018;103:155–164; DOI: 10.1136/archdischild-2017-313375) has been corrected and republished. The previous text of the article, marked up to show all the changes made, has been posted as an online supplementary file to the republished article.

10.1136/archdischild-2017-313375corr1.supp1 Supplementary data

The changes are extensive but clarificatory and for this reason the editors have issued a correction and not a retraction. A linked Editor’s note provides more background to this decision (10.1136/archdischild-2017-313375ednote).

The corrections include, but are not limited to, the following. (All corrections are shown on the online marked version, which is free to access.) Some changes to punctuation and reference numbers have not been noted below but are included in the marked copy.

## Abstract

‘SF-36-PFS at three and 12 months’ was added to the list of secondary outcomes in the ‘Main outcome measures’ section. In the Results section ‘We recruited 100 participants, of whom 51 were randomised to SMC+LP.’ has been changed to ‘We recruited 100 participants between September 2010 and September 2013. We tested the feasibility of running the trial with a feasibility phase (29th September 2010 to 18th September 2012). The full trial was registered in June 2012 when we had determined it was a feasible study. Of the 100 participants, 51 were randomised to SMC+LP.’ In the Results section, ‘probably’ has been deleted and the p value changed to 0.03 in the following line: ‘SMC+LP was probably more cost effective in the multiple imputation dataset (difference in means in net monetary benefit at 12 months £1474, [95% CI £111 to £2836], p=0.034) but not for complete cases.’

## Introduction

Paragraph 1: ‘affects’ changed to ‘effects’. Paragraph 3: ‘LP is not available in the NHS.’ has been deleted.

### Methods, Study design and participants

‘Between September 2010 and April 2013, we recruited participants after clinical assessment by the Bath/Bristol paediatric CFS/ME service, a large regional and national NHS specialist service.’ has been changed to read ‘Between September 2010 and September 2013 we recruited participants after clinical assessment by the Bath/Bristol paediatric CFS/ME service, a large regional and national NHS specialist service. We tested the feasibility of running this trial with a feasibility phase (29th September 2010 to 18th September 2012). We determined the trial was feasible in June 2012 and registered the full trial (31st July 2012). We applied for an amendment to recruit children into the full trial as opposed to a feasibility trial (see web table 1 for detailed description of amendments). Full trial first randomisation was the 19th September 2012. We continued seamlessly with participant recruitment without any interim between-group comparison of participant outcome data from the feasibility phase. Children from both phases (feasibility and full) were analysed.’

### Methods, Randomisation and masking

‘the randomised intervention was conveyed during the recruitment interview’ has been changed to ‘the randomised intervention was conveyed after obtaining consent, during the recruitment interview’.

### Methods, Interventions

‘Sessions were delivered by a range of trained and supervised professionals including doctors…’ has been changed to read ‘Sessions were delivered by a range of professionals including doctors…’.

The following paragraph was removed: ‘Lightning Process practitioners have completed a Diploma through the Phil Parker Training Institute in Neurolinguistic Programming, Life Coaching and Clinical Hypnotherapy. This diploma is examined through written and practical exams and is accredited by the British Institute of Hypnotherapy and NLP. Following the Diploma, Lightning Process practitioners undertake a further course to learn the tools and delivery required for the Lightning Process after which they must pass both a practical and written exam. Practitioners undertake supervision and CPD in order to further develop their skills and knowledge. They are regulated by the Register of Lightning Process practitioners, adhere to a Code of Conduct, and there is a Professional Conduct Committee that oversees complaints and professional practice issues.’

### Methods, Outcomes

The first paragraph as published read:

‘The primary outcome was the the 36-Item Short-Form Health Survey Physical Function Subscale (SF-36-PFS)^26^ analysed as a continuous variable collected at 6 month post-randomisation. Secondary outcomes were the SF-36-PFS at 3 and 12 months, and school attendance (days per week), the Chalder Fatigue Scale^27^ and quality-adjusted life years (QALYs, derived from the EQ-5D-Y)^28^ at 3, 6 and 12 months. Pain was measured by a Visual Analogue Scale (VAS) at 6 months. All were self-completed by participants. Participants also completed the Hospital Anxiety and Depression Scale (HADS)^29^ and the Spence Children’s Anxiety Scale (SCAS)^30^ at assessment, and at 3, 6 and 12 months. At 3, 6 and 12 months, parents completed an adapted four-item Work Productivity and Activity Impairment: General Health V2.0 questionnaire (V2.0)^31^ and a resource use questionnaire assessing their child’s health service use (eg, general practitioner or specialist care), educational service use (eg, school counsellor), health-related travel and other family costs.’

­

This has been expanded to read as follows:

‘The primary outcome was the SF-36 physical function subscale (SF-36-PFS^26^) analysed as a continuous variable collected at 6 months post-randomisation. We chose the SF-36 based on qualitative work conducted in the feasibility phase of the study.^24^ We have reported that parents and participants ‘commented that the school attendance primary outcome did not accurately reflect what they were able to do, particularly if they were recruited during, or had transitioned to, A levels during the study’. In addition, ‘we were aware of some participants who had chosen not to increase school attendance despite increased activity.’ We therefore concluded that: “trials involving 17 and 18 year olds should consider alternative primary outcome measures to school attendance as it is difficult to assess for those transitioning from GCSEs to A levels, and may not be appropriate for those who do not consider school attendance their primary goal’. At this stage, our recommendation was that a “full study uses other primary outcomes, such as the SF-36 or the Chalder Fatigue Scale and uses school attendance as a secondary outcome.’ These findings informed our application for our ethical amendment to a full study in 2011 (see web table 1). And were published in our feasibility paper in 2013.^24^

Qualitative interviews with SMILE participants then formed part of a larger study which described the conceptual model for paediatric CFS/ME.^27^ In this study, physical activity (or disability) is described by children as being pivotal because of the impacts on social participation and emotional well-being. While school was deemed to be an important contextual factor, these qualitative results led us to choose the SF-36-PFS as a primary outcome with school attendance as a secondary outcome. There was no analysis of any outcome data during or after the feasibility phase until the entire trial was completed.

Secondary outcomes were the SF-36-PFS at three and 12 months, and school attendance (days per week), the Chalder Fatigue scale^28^, pain (visual analogue scale), Hospital Anxiety and Depression Scale (HADS)^29^, Spence Children’s Anxiety Scale (SCAS)^30^ and quality-adjusted life years (QALYs, derived from the EQ-5D-Y)^31^ at three, six and 12 months. At three, six and 12 months parents completed an adapted four item Work Productivity and Activity Impairment: General Health V2.0 (WPAI:GH) questionnaire (V2.0)^32^ and a resource use questionnaire assessing their child’s health service use (eg, GP or specialist care), educational service use (eg, school counsellor), health related travel and other family costs.’

### Methods, Sample size

This paragraph originally read:

‘We used a consensus definition for a small clinically important difference of 10 points on the SF-36-PFS.^32^ Thirty two to 50 participants in each arm are required to detect a between-group difference of 8 to 10 points on the SF-36-PFS (SD 10) at 6 months with 90% power and 1% two-sided significance. Allowing for 10% to 20% non-collection of primary outcome data, we aimed to recruit 80 to 112 participants.’

It has been changed to read:

‘A consensus definition for a small clinically important difference on the SF-36-PFS at 6 months follow-up is 10 points.^33^ However, we did not want to miss a smaller but still potentially important effect of as low as eight points. To detect a between-group difference of 8 to 10 points with 90% power, 1% two-sided significance and SD of 10 requires between 32 and 50 participants per group for analysis. Allowing for 10% to 20% non-collection of primary outcome data, we aimed to recruit between 80 (32*2/0.8) and 112 (50*2/0.9) participants.’

### Methods, statistical analysis

First paragraph: ‘Sensitivity analyses of the primary outcome adjusted for variables for which there was baseline imbalance; excluded those recruited up to 31 January 2011 preceding the protocol amendment; and used multiple imputation of missing data (see online supplementary appendix 1 for details).’ now reads ‘Sensitivity analyses of the primary outcome adjusted for variables for which there was baseline imbalance; excluded those recruited up to 31 January 2011 preceding the protocol amendment to allow collection of follow-up data by phone; and used multiple imputation of missing data.’ The line ‘Twelve-month outcome data were analysed similarly.’ has been deleted. The line ‘We did not analyse 3 month outcomes except in this repeated measures analysis for SF-36-PFS as these were unlikely to be informative since the primary follow-up was at 6 months.’ has been added.

### Methods, Health economic analyses

Some references have been renumbered, as have some references to online supplementary material. The penultimate paragraph has had a further reference to online supplementary material added.

## Results

First paragraph: the text ‘56 of these participants were included in the report of whether it was feasible to conduct this RCT.^24^’ was added before ‘Recruitment was stopped after the 100th participant was randomised.’

Second paragraph: the sentence ‘The imbalance in pain and SCAS scores were in opposite directions suggesting that the two arms were not systematically different.’ was added. Some online supplementary tables were renumbered. ‘Three participants in the SMC+LP arm received the LP course after completing the 6 month follow-up.’ was replaced by ‘Three participants (3/39, 8%) in the SMC+LP arm received the LP course after completing the 6 month follow-up, these participants were included in the analyses.’

Third paragraph: ‘The average between-arm difference in physical function across both 6 and 12 month follow-up was 14.4 (95% CI 7.3 to 21.5), p<0.001. The estimated effect of LP (using CACE analyses) among compliers at 6 and 12 months was increased compared with the intention-to-treat (ITT) estimate (table 2).’ was replaced by ‘The average between-arm difference in physical function across both 6 and 12 month follow-up was 14.4 (95% CI 7.3, 21.5, p<0.001). When compliance was taken into account using CACE analyses, the estimated effect of LP 6 and 12 months was increased compared with the ITT estimate (table 2).’

Fourth paragraph, first sentence: ‘p=0.039’ was replaced by ‘p=0.04’; second sentence: the word ‘somewhat’ has been deleted from ‘The difference in means in fatigue score and HADS anxiety score were somewhat smaller at 12 months’; third sentence: ‘p=0.030’ replaced by ‘p=0.03’; fourth sentence: ‘p=0.18’ replaced by ‘p=0.2’; fifth sentence: ‘Pain scores were lower’ replaced by ‘Mean pain scores were lower’.

Sixth paragraph: ‘p=0.276’ replaced by ‘p=0.03’.

Seventh paragraph: ‘Complete healthcare use questionnaires were returned by between 55 (55% at 12 months) and 56 (56% at 3 and 6 months) participants, but only 30 (30%) completed these questionnaires at all three time points (see online supplementary table S5 for details).’ changed to ‘Complete healthcare use questionnaires were returned by 56 participants at 3 and 6 months and 55 at 12 months, but only 30 participants completed these questionnaires at all three time points.’ In the final sentence the p values 0.082 and 0.000 have been replaced by 0.08 and <0.005 respectively.

Eighth paragraph: p value 0.034 has been changed to 0.03; references to online supplementary material have been added; the sentence ‘Sensitivity analyses assuming costs and QALYs are not missing at random^40^ did not alter the conclusion that SMC+LP was likely to be cost-effective, but reduced the strength of the evidence.’ now reads ‘Sensitivity analyses assuming costs and QALYs are not missing at random^40^ reduced the strength of the evidence that SMC+LP was likely to be cost-effective, but did not alter the conclusion.’

## Discussion

Paragraph one has been amended from ‘Participants in the LP arm were attending 1 day more of school a week at 12 months’ to read ‘Participants in the LP arm were attending 1 day more of school a week at 12 months on average.’ Reference 40 was changed to 41.

Paragraph two: ‘that we followed patients’ was changed to ‘follow’. The following sentence was added: ‘We did not have capacity to check school attendance using school records, but this could have provided an objective outcome. Further unpublished work suggests this is highly correlated with the self report measure we used.’ The following was added to the end of paragraph two: ‘The study was originally planned as a feasibility study and although randomised, was not registered on a trial registry, since the aim was to investigate feasibility rather than effectiveness of the intervention. After establishing feasibility, we applied to register the full trial in June 2012. At this time, the results of our feasibility work suggested we could use either SF-36-PFS or Chalder Fatigue scale or both which we registered as primary outcomes. We decided to use just the SF-36-PFS and published this in 2013 and in our analyses plan. We did not update the ISRCTN site until 2018, however, we uploaded the relevant publications in 2016 and the study website had the updated analyses plan.’

Additional sentences were added to the end of paragraph three: ‘The study was registered after demonstrating feasibility. The analysis includes participants who were recruited prior to registration of the study. This does not comply with ICMJE and BMJ guidance on trial registration. The reasons for this have been explained in the paper.’

The following section was added to paragraph four:

‘We pre-defined the minimally clinically important difference as 10 points on the SF-36-PFS based on consensus statements. Subsequent work by our team has shown that this is a clinically significant change in physical function for children with CFS/ME.^42^ Ten points equates to a minimum of two step changes on the SF-36-PFS. This can be either one step change on two questions, or two step changes on one question. The SF-36 asks: ‘Does your health limit you in these activities? If so, how much? As an example, one step change could be: ‘Yes, limited a lot’ to ‘Yes, limited a little’ or ‘Yes, limited a little’ to ‘No, not limited at all’ to different questions such as ‘climbing several flights of stairs’ or ‘walking 100 yards’ or ‘walking half a mile’.’

The last line of the paragraph (‘As we did not compare LP with either a full course of only CBT or GET we do not know if LP is more of less effective than either of these treatment approaches.’) was deleted.

A new section, ‘Conclusions’, was added.

## Acknowledgements

The statement ‘No member of the LP team had any involvement in the analyses or in writing the paper.’ has been moved to the Disclaimer section.

## Contributors

The lines ‘The authors had access to all the data. The corresponding author had full access to all the data in the study and had final responsibility for the decision to submit for publication.’ have been moved from the Data sharing statement to the Contributors section.

## Disclaimer

The following lines were added to the Disclaimer: ‘The funders and the sponsor of the study had no role in the design and conduct of the study; collection, management, analysis and interpretation of the data; preparation, review or approval of the manuscript; and decision to submit the manuscript for publication. All researchers involved in this study were independent from both the funders and the sponsor.’

## Competing interests

This section has been expanded to read as follows: ‘Financial support for the submitted work was received from the Linbury Trust and the Ashden Trust. EMC and SMC have received fellowship grants from the NIHR. JACS has received grants from the NIHR. EMC runs the specialist CFS/ME service at Royal United Hospital NHS Foundation Trust, has received one grant from MRC, one grant from the NIHR and is a medical advisor to the Sussex and Kent ME/CFS Society. The authors declare they did not receive any funding from the Lightning Process.’

## Ethics approval

This section has been expanded to read as follows: ‘A favourable ethical opinion was given on 8 September 2010 (reference 10/H0206/32) by South West 2 Local Research Ethics Committee. Two favourable opinions were provided for amendments to study documents and protocol on 31 May 2011 and 6 September 2012.’

## Tables

Changes have also been made to tables 1–4; these are shown in the marked copy and relate to rounding up some entries and adding information to the legends.

## References

A new reference 27 has been added (Parslow *et al*). The previous reference 28 (Ravens-Sieberer *et al*) is now reference 31. Subsequent references have been renumbered. A new reference (Brigden *et al*) has been added.

## Competing interests

Additional sentence: ‘The authors declare they did not receive any funding from the Lightning process.’

