# Editor’s note on correction to Crawley *et al. (2018)*

**DOI:** 10.1136/archdischild-2017-313375ednote

**Published:** 2019-07-11

**Authors:** Nick Brown

**Affiliations:** 1 Department of Women’s and Children’s Health, International Maternal and Child Health (IMCH), Uppsala University, Uppsala, Sweden; 2 Department of Paediatrics, Länssjukhuset Gävle-Sandviken, Gävle, Sweden; 3 Department of Child Health, Aga Khan University, Karachi, Pakistan

**Crawley EM, Gaunt DM, Garfield K, *et al*. Clinical and cost-effectiveness of the Lightning Process in addition to specialist medical care for paediatric chronic fatigue syndrome: randomised controlled trial. *Arch Dis Child* 2018;103:155–64. doi: 10.1136/archdischild-2017-313375.**

This trial (ISRCTN81456207: registration July 31 st 2012) was originally submitted to *Archives of Disease in Childhood* (*ADC*) in May 2017 and underwent external peer review. It was accepted after one revision on July 28^th^ 2017, was published online first on September 20^th^ 2017 and in print in the February 2018 edition (Volume 103, issue 2).

The study tested the effectiveness of the Lightning Process, a neurolinguistic programming intervention used widely but never formally tested, in children and young people with chronic fatigue syndrome (CFS) recruited between 2010 and 2013. Though the number of participants was small and the enrolment rate low, analysis suggested a benefit in terms of physical function (measured by the standard SF 36 scale) at both 6 and 12 months after intervention. Secondary analysis indicated that the treatment was likely to be cost effective.

In January 2018, *ADC* was contacted by a group of readers (http://www.virology.ws/2018/01/30/trial-by-error-a-letter-to-archives-of-disease-in-childhood/) with specific criticisms of the paper. The authors were criticised for a lack of clarity with respect to International Committee of Medical Journal Editors (ICMJE) and BMJ policies on trial registration (in place since 2007) and for not fully adhering to CONSORT guidance on trial reporting.^1 2^ The journal was criticised for not detecting these issues during the review process.

The issues related to the following areas:

1. The inclusion of children from the feasibility phase (recruited between October 2010 and July 2012) in the analysis of the main trial. The authors sought approval for the change from the relevant ethics committee (June 2012), but, the chronology was not clear in the original paper. In part this was because no timeline was provided and in part because the tense used in the text implied that recruitment had been purely prospective. The ISRCTN entry (dated July 2012) stated: ‘the study started as a feasibility study in October 2010’. In the text, for example the authors stated that ‘young people and parents will be asked to read the information’ and that ‘we have now shown that it is possible to run the study and are planning to convert the study to a randomised trial’ suggesting that recruitment of eligible participants had not started in June 2012. The editorial and review processes did not pick up these issues with the timeline at this stage.
2. A concern that the primary outcomes were “swapped” between the feasibility and full trials. The authors’ feasibility study publication explains why the 36-Item Short-Form Health Survey Physical Function Subscale (SF-36-PFS) was selected as the primary outcome for the full trial, after school attendance had been considered as a potential primary outcome in the feasibility study, but this was not explained in the article. The SF-36-PFS was also included as the primary outcome in the published protocol for the full trial.

When these criticisms were raised with the journal, we acknowledged them as valid and embarked on a lengthy clarification process with the authors. To do so with the appropriate rigour has taken over a year and the chronology is worth detailing.

1. January 2018: Criticisms of the paper received
2. February 2018: An external review of the chronology to establish the validity of the criticisms and confirmation that substantial clarification of the above issues would be needed
3. March 2018: Feedback of detailed comments to the authors with instructions as to the prerequisites for the clarified paper
4. June 2018: The posting of an e-letter linked to the paper alluding to steps being taken to address the criticisms (https://adc.bmj.com/content/103/2/155.responses)
5. July 2018: First version of the corrected paper received. Sent for statistical review and review by an experienced *ADC* editor not involved with handling the article previously
6. July 2018: Reviewers’ and editors’ comments sent to authors
7. October 2018: First revision of the paper received. Sent for re-review by previous reviewers
8. November 2018: Reviewers’ and editors’ comments sent to authors
9. November 2018: Second revision received and, after further discussion and editorial review, provisionally accepted in February 2019
10. March 2019: Formal acceptance of revised paper.

The final version includes acknowledgement from the authors that the study was not fully ICMJE compliant. The process has additionally involved seeking assurance from the authors that the change in primary outcome was not influenced by (positive) findings in the feasibility phase.

We are now satisfied that the paper is a robust account of events and that it can be considered as a contribution to the field of CFS research.

We agree with the correspondents that the article as originally published lacked sufficient detail and clarity for readers to fully understand the study as conducted. Therefore we have published a substantial correction^3^ which adds extensive clarifications to the study’s timeline and methods.

We have taken the step of republishing the article with the clarifications incorporated because they are so extensive. This republished article links to a detailed note of correction and clarification and we have also posted a breakdown of every change to the article, to ensure transparency (10.1136/archdischild-2017-313375corr1 and 10.1136/archdischild-2017-313375).

BMJ policy requires prospective registration of randomised trials but we do not consider a failure to enforce that policy grounds for retraction. The corrections to the article are mainly clarifications and we do not believe the article meets any of the Committee on Publication Ethics’ criteria for retraction.

In addition, we have revised our processes in relation to EQUATOR-CONSORT guidance to ensure robustness. As is clear on our instructions to authors, no trial in which recruitment predates trial registration will be considered further.

We are satisfied that the correction this manuscript has undergone has addressed the criticisms raised and we are grateful to the correspondents for bringing them to our attention.
