# Supplementary material for: Clinical and cost-effectiveness of the Lightning Process in addition to specialist medical care for paediatric chronic fatigue syndrome: randomised controlled trial
Source: Arch Dis Child. 2017 Sep 20;103(2):155–64. doi: 10.1136/archdischild-2017-313375 (PMC5865512; doi:10.1136/archdischild-2017-313375)
Supplement: Supplementary file 1 [file archdischild-2017-313375supp001.docx]

**Web table 1 – Unit costs used for economic evaluation**

| Cost category | Resource | Unit cost (£) | Source of unit cost |
| --- | --- | --- | --- |
| Lightning Process | Trial course cost, mean contact hours: 13·42 | 567* | Phil Parker Lightning Process |
|  | National course cost, mean contact hours: 13·42 | 620* |  |
|  | NHS course cost, mean contact hours: 12·34 | 444** | Unit costs of Health & Social Care^35^, NHS Agenda for Change^41^ |
| Standard Medical Care | Consultant, first | 223 | Department of Health (DH) reference costs^34^ |
|  | Consultant, follow-up | 172 |  |
|  | Consultant, telephone | 115 |  |
|  | Consultant psychologist, first | 264 |  |
|  | Consultant psychologist, follow-up | 233 |  |
|  | Consultant psychologist, telephone | 15 |  |
|  | Non-consultant psychologist, first | 251 |  |
|  | Non-consultant psychologist, follow-up | 192 |  |
|  | Non-consultant psychologist, telephone | 34 |  |
|  | Occupational therapist, first | 76 |  |
|  | Occupational therapist, follow-up | 60 |  |
|  | Occupational therapist, telephone | 39 |  |
|  | Physiotherapist, first | 51 |  |
|  | Physiotherapist, follow-up | 39 |  |
|  | Physiotherapist, telephone | 30 |  |
| Hospital services | Hospital outpatient clinic | 108 | DH reference costs^34^ |
|  | A&E | 115 |  |
|  | Other hospital visits | By item |  |
| Primary and community care | GP, consultation | 45 | Unit costs of health and social care^35^ |
|  | Nurse, consultation | 17 |  |
|  | GP, telephone | 27 |  |
|  | Nurse, telephone | 10 |  |
|  | GP, home visit | 114 |  |
|  | School counsellor | 164 | Hill et al^37^ |
|  | Walk-in-centre nurse | 43 | DH reference costs^34^ |
|  | NHS direct, telephone | 29 | Parliament publication^46^ |
|  | Other Primary & Community care | By item |  |
| Prescribed medication | | By item | Prescription Cost Analysis^36^ |
| Personal costs | Additional spending on child | Self-report |  |
| Productivity | Loss of earnings, past 3 months | Self-report |  |
|  | Loss of earnings, median hourly earnings | 13·03 | Office for National Statistics^47^ |
| *Price charged for all (n=42) participants who attended at least 1 day of the course: 3 participants only attended 1 day. | | | |
| **Mean estimated cost for all (n=46) participants who had any contact. We estimated the cost of NHS practitioners providing LP assuming they would be a mid-Band 7 with supervision from a mid-Band 8a. Standard unit costs (including overheads) for practitioner time^35^ were applied and adjusted to reflect mid-Band 7 and mid-Band 8a salaries ^45^. | | | |

**Web table 2: Baseline characteristics of those who found out more about the study but were not randomized compared to the randomized population**

|  | **Eligible but not randomized** | | **Randomized** | |
| --- | --- | --- | --- | --- |
|  |  | **N** |  | **N** |
| **Demographic characteristics** |  |  |  |  |
| Mean age (SD) | 14·9 (1·6) | 31 | 14·6 (1·5) | 100 |
| Number female (%) | 22 (71·0%) | 31 | 76 (76·0%) | 100 |
| Median months from onset of illness to baseline assessment (25^th^ percentile, 75^th^ percentile) | 12·0 (7·5, 17·0) | 20 | 12·0 (8·0, 20·0) | 98 |
|  |  |  |  |  |
| **Clinical characteristics** |  |  |  |  |
| Mean SF-36 physical function score^1^ (SD) | 58·2 (27·2) | 30 | 54·5 (20·2) | 99 |
| Mean Chalder Fatigue score^2^ (SD) | 24·4 (5·1) | 31 | 25·0 (4·2) | 99 |
| Mean pain VAS^2^ (SD) | 49·4 (33·1) | 27 | 47·0 (29·2) | 96 |
| Mean SCAS^2^ (SD) | 25·7 (19·7) | 29 | 35·0 (19·2) | 97 |
| Mean HADS Anxiety score^2^ (SD) | 8·0 (5·2) | 28 | 9·6 (4·5) | 99 |
| Mean HADS Depression score^2^ (SD) | 6·1 (3·4) | 28 | 7·8 (3·8) | 98 |
| Mean EQ-5D score^1^ (SD) | 0·34 (0·40) | 22 | 0·33 (0·35) | 100 |
| School attendance in the previous week^1^ N (%): |  |  |  |  |
| None | 7 (21·9%) | 32 | 13 (13·1%) | 99 |
| 0·5 day | 2 (6·3%) | 32 | 12 (12·1%) | 99 |
| 1 day | 1 (3·1%) | 32 | 6 (6·1%) | 99 |
| 2 days | 4 (12·5%) | 32 | 16 (16·2%) | 99 |
| 3 days | 5 (15·6%) | 32 | 24 (24·2%) | 99 |
| 4 days | 10 (31·3%) | 32 | 21 (21·2%) | 99 |
| 5 days | 2 (6·3%) | 32 | 7 (7·1%) | 99 |
| N/A | 1 (3·1%) | 32 | 0 (0·0%) | 99 |
| HADS: Hospital Anxiety and Depression Scale; IQR: Interquartile range; SCAS: Spence Children’s Anxiety Scale; SD: Standard deviation; SF-36: The 36-item short-form health survey; VAS: Visual Analogue Scale. All results rounded to 1 d.p. ^1^Higher score=fewer symptoms, better function. ^2^Higher score=more symptoms, poorer function. | | | | |

**Web Table 3: Baseline** **characteristics of the randomized population who completed or did not complete primary outcome at 6 months**

|  | **Completed SF-36 Physical Function** | | **Did not complete SF-36 Physical Function** | |
| --- | --- | --- | --- | --- |
|  |  | **N** |  | **N** |
| **Demographic characteristics** |  |  |  |  |
| Mean age (SD) | 14·6 (1·6) | 82 | 14·4 (1·3) | 18 |
| Number female (%) | 65 (79·3 %) | 82 | 11 (61·1%) | 18 |
| Median months from onset of illness to baseline assessment (25^th^ percentile, 75^th^ percentile) | 12·0 (8·0, 21·0) | 81 | 12·0 (8·0, 18·0) | 17 |
|  |  |  |  |  |
| **Clinical characteristics** |  |  |  |  |
| Mean SF-36 physical function score^1^ (SD) | 54·0 (20·9) | 81 | 56·9 (16·8) | 18 |
| Mean Chalder Fatigue score^2^ (SD) | 25·2 (4·3) | 81 | 24·4 (3·9) | 18 |
| Mean pain VAS^2^ (SD) | 47·0 (29·5) | 78 | 47·2 (28·7) | 18 |
| Mean SCAS^2^ (SD) | 35·3 (19·4) | 81 | 33·4 (18·8) | 16 |
| Mean HADS Anxiety score^2^ (SD) | 9·7 (4·7) | 81 | 9·3 (3·7) | 18 |
| Mean HADS Depression score^2^ (SD) | 7·7 (3·7) | 80 | 8·4 (4·2) | 18 |
| Mean EQ-5D score^1^ (SD) | 0·33 (0·36) | 82 | 0·29 (0·31) | 18 |
| School attendance in the previous week^1^ N (%): |  |  |  |  |
| None | 11 (13·6%) | 81 | 2 (11·1%) | 18 |
| 0·5 day | 8 (9·9%) | 81 | 4 (22·2%) | 18 |
| 1 day | 3 (3·7%) | 81 | 3 (16·7%) | 18 |
| 2 days | 14 (17·3%) | 81 | 2 (11·1%) | 18 |
| 3 days | 22 (27·2%) | 81 | 2 (11·1%) | 18 |
| 4 days | 17 (21·0%) | 81 | 4 (22·2%) | 18 |
| 5 days | 6 (7·4%) | 81 | 1 (5·6%) | 18 |
| HADS: Hospital Anxiety and Depression Scale; IQR: Interquartile range; SCAS: Spence Children’s Anxiety Scale; SD: Standard deviation; SF-36: The 36-item short-form health survey; VAS: Visual Analogue Scale. All results rounded to 1 d.p. ^1^Higher score=fewer symptoms, better function. ^2^Higher score=more symptoms, poorer function. | | | | |

**Web table 4: Subgroup analysis of SF-36 physical function at 6 months**

|  | **SMC group** | | **SMC plus LP group** | | **Difference in means ^1^**  **(95% CI)** | **N** | **Adjusted interaction^1 2^**  **(95% CI)** | **N** | **P-value** |
| --- | --- | --- | --- | --- | --- | --- | --- | --- | --- |
|  | **Mean** | **N** | **Mean** | **N** |  |  |  |  |  |
| Children <15 years | 70·8 | 19 | 83·9 | 19 | 13·8 (2·1, 25·5) | 38 | -2·8 (-19·0, 13·4) | 81 | 0·734 |
| Children 15 to <18 years | 69·6 | 18 | 80·0 | 26 | 11·0 (0·14, 21·9) | 43 |  |  |  |
| Children male | 67·5 | 8 | 86·7 | 9 | 26·6 (8·9, 44·3) | 17 | -17·6 (-37·3, 2·1) | 81 | 0·080 |
| Children female | 70·9 | 29 | 80·4 | 36 | 9.0 (0.2, 17.8) | 64 |  |  |  |
| Children no school/college attendance at baseline | 67·4 | 5 | 85·0 | 6 | 23·3 (1·2, 45·5) | 11 | -12·0 (-35·9, 12·0) | 80 | 0·323 |
| Children some school/college attendance at baseline | 70·6 | 32 | 82·1 | 38 | 11.4 (2.7, 20.0) | 69 |  |  |  |
| Children without co-morbid anxiety (<12 HADS Anxiety) at baseline | 71·6 | 22 | 80·1 | 30 | 10·9 (0·6, 21·1) | 52 | 4·8 (-12·5, 22·1) | 80 | 0·579 |
| Children with co-morbid anxiety (≥12 HADS Anxiety) at baseline | 69·4 | 14 | 84·7 | 15 | 15·7 (1·9, 29·5) | 28 |  |  |  |
| Higher score=fewer symptoms, better function. ^1^Interaction represents SMC plus LP minus SMC in subgroup 2 minus SMC plus LP minus SMC in subgroup 1. ^2^Adjusted for age, gender and baseline outcome. | | | | | | | | | |

**Web table 5: Health care use at 3, 6 and 12 months in complete cases; by treatment group**

|  |  | **SMC** | | | | | **SMC plus LP** | | | | | **Difference in mean cost (95% CI)** | | **N** | **Adjusted difference in mean cost* (95% CI)** | | **N** |
| --- | --- | --- | --- | --- | --- | --- | --- | --- | --- | --- | --- | --- | --- | --- | --- | --- | --- |
|  |  | **Mean contacts** | **(SD)** | **Mean cost** | **(SD)** | **N** | **Mean contacts** | **(SD)** | **Mean cost** | **(SD)** | **N** |  |  |  |  |  |  |
| 0 - 3 Months | |  |  |  |  |  |  |  |  |  |  |  |  |  |  |  |  |
|  | Lightning Process |  |  | 12 | (81) | 49 |  |  | 211 | (277) | 51 | 200 | (118, 282) | 100 |  |  |  |
|  | Outpatient | 3.1 | (1.3) | 412 | (266) | 49 | 3.1 | (1.3) | 449 | (267) | 51 | 36 | (-70, 142) | 100 | 21 | (-96, 138) | 89 |
|  | Other Hospital** | 0.2 | (0.9) | 27 | (97) | 23 | 0.3 | (0.8) | 32 | (73) | 34 | 5 | (-40, 0) | 57 | 6 | (-37, 50) | 53 |
|  | Primary care*** | 3.2 | (4.3) | 126 | (161) | 25 | 3.3 | (5.4) | 113 | (189) | 34 | -13 | (-106, 81) | 59 | -32 | (-120, 57) | 54 |
|  | Other Community**** | 0.3 | (0.7) | 13 | (50) | 25 | 0.8 | (1.5) | 26 | (120) | 34 | 13 | (-38, 65) | 59 | -4 | (-22, 13) | 54 |
|  | School counsellor | 0.1 | (0.4) | 13 | (66) | 25 | 0.6 | (1.8) | 92 | (303) | 34 | 79 | (-45, 202) | 59 | 96 | (-52, 244) | 54 |
|  | Prescribed medication |  |  | 25 | (57) | 25 |  |  | 34 | (69) | 35 | 8 | (-25, 42) | 60 | -10 | (-35, 14) | 56 |
|  | Total cost including LP |  |  | 590 | (429) | 23 |  |  | 949 | (534) | 33 | 358 | (89, 628) | 56 | 303 | (133, 473) | 51 |
| 4 - 6 Months | |  |  |  |  |  |  |  |  |  |  |  |  |  |  |  |  |
|  | Lightning Process |  |  | 12 | (81) | 49 |  |  | 234 | (282) | 51 | 222 | (139, 305) | 100 |  |  |  |
|  | Outpatient | 1.6 | (1.2) | 237 | (225) | 49 | 1.7 | (1.4) | 221 | (189) | 51 | -15 | (-98, 67) | 100 | -45 | (-141, 50) | 89 |
|  | Other Hospital** | 0.5 | (1.6) | 87 | (340) | 26 | 0.3 | (0.7) | 28 | (84) | 32 | -59 | (-184, 66) | 58 | 16 | (-20, 51) | 54 |
|  | Primary care*** | 1.6 | (2.5) | 61 | (107) | 25 | 1.3 | (2.1) | 44 | (74) | 32 | -17 | (-65, 31) | 57 | -28 | (-72, 16) | 52 |
|  | Other Community**** | 0.2 | (0.5) | 33 | (123) | 25 | 0.3 | (0.4) | 4 | (17) | 32 | -29 | (-73, 15) | 57 | -27 | (-81, 28) | 52 |
|  | School counsellor | 0.3 | (1.1) | 52 | (175) | 25 | 0.2 | (0.5) | 26 | (84) | 32 | -27 | (-97, 44) | 57 | 1 | (-79, 80) | 52 |
|  | Prescribed medication |  |  | 17 | (41) | 26 |  |  | 47 | (114) | 33 | 30 | (-17, 77) | 59 | 15 | (-27, 56) | 56 |
|  | Total cost including LP |  |  | 570 | (599) | 25 |  |  | 620 | (476) | 31 | 50 | (-238, 338) | 56 | 205 | (-25, 435) | 50 |
| 7 - 12 Months | |  |  |  |  |  |  |  |  |  |  |  |  |  |  |  |  |
|  | Lightning Process |  |  | 12 | (81) | 49 |  |  | 22 | (111) | 51 | 11 | (-28, 49) | 100 |  |  |  |
|  | Outpatient | 2.0 | (1.6) | 297 | (336) | 49 | 1.8 | (2.1) | 242 | (338) | 51 | -56 | (-189, 78) | 100 | -85 | (-231, 61) | 89 |
|  | Other Hospital** | 0.1 | (0.3) | 12 | (43) | 26 | 0.3 | (0.9) | 65 | (214) | 32 | 54 | (-32, 139) | 58 | 59 | (-43, 162) | 52 |
|  | Primary care*** | 2.5 | (3.8) | 101 | (154) | 25 | 2.7 | (4.1) | 90 | (140) | 30 | -10 | (-90, 69) | 55 | -33 | (-103, 37) | 47 |
|  | Other Community**** | 0.2 | (0.4) | 25 | (115) | 25 | 0.8 | (1.7) | 40 | (210) | 30 | 15 | (-79, 110) | 55 | -10 | (-62, 42) | 47 |
|  | School counsellor | 0.2 | (0.8) | 39 | (136) | 25 | 0.0 | (0.2) | 27 | (122) | 30 | -12 | (-82, 58) | 55 | -2 | (-92, 87) | 47 |
|  | Prescribed medication |  |  | 18 | (50) | 26 |  |  | 32 | (106) | 32 | 14 | (-32, 59) | 58 | 0 | (-39, 39) | 52 |
|  | Total cost including LP |  |  | 617 | (560) | 25 |  |  | 464 | (637) | 30 | -154 | (-481, 174) | 55 | -302 | (-482, -122) | 47 |
| Total 12 Month cost including LP | | |  | 1388 | (1039) | 12 |  |  | 1802 | (1045) | 18 | 414 | (-382, 1210) | 30 | 445 | (-148, 1038) | 27 |
| *adjusted for baseline difference, age sex, baseline SCAS and baseline VAS | | | | | | | | | | |  |  |  |  |  |  |  |
| ** Other hospital includes A&E, CAMHs and other hospital visits | | | | | | | | | |  |  |  |  |  |  |  |  |
| *** Primary care includes GP and Nurse appointments, calls and home visits, walk-in-care and calls to NHS direct | | | | | | | | | | | | | |  |  |  |  |
| **** Other community includes school nurse, CAMHs, dietician, etc. | | | | | | | | | | | | |  |  |  |  |  |

**Web table 6: Sensitivity analyses: Variations of the cost of LP in multiple imputation dataset**

|  | **SMC (n=49)** | | **LP plus SMC (n=51)** | | **Incremental difference** | |
| --- | --- | --- | --- | --- | --- | --- |
|  | **Mean (SE)** | | **Mean (SE)** | | **(95% CI)** | |
| **Imputed 12 Month - LP national cost**** |  |  |  |  |  |  |
| Adjusted total cost (£) | 1615 | (85) | 2045 | (67) | 430 | (228, 632) |
| Adjusted QALYs | 0·533 | (0·025) | 0·628 | (0·021) | 0·095 | (0·030, 0·160) |
| NMB at £20,000 per QALY | 9039 | (521) | 10508 | (427) | 1468 | (108, 2829) |
| **Imputed 12 Month - LP NHS cost***** |  |  |  |  |  |  |
| Adjusted total cost (£) | 1604 | (84) | 1935 | (67) | 331 | (130, 531) |
| Adjusted QALYs | 0·533 | (0·025) | 0·628 | (0·021) | 0·095 | (0·030, 0·160) |
| NMB at £20,000 per QALY | 9050 | (521) | 10618 | (427) | 1568 | (207, 2929) |
| *All adjusted for baseline value, sex, age, baseline SCAS and baseline VAS | | | | | | |
| **National cost equals current average cost charged for LP | | | | | | |
| ***NHS cost is estimated using LP contact time and relevant unit costs | | | | | | |

**Web Appendix 1: Multiple imputation methods**

Multiple imputation by chained equations (*ice* procedure^48^ version 1.9.7 dated 25/10/2014) was used to impute missing data for 50 datasets. The imputation model included age, gender, SF-36-PFS, Chalder Fatigue score, VAS, SCAS, EQ-5D-Y, questionnaire costs and outpatient costs based on hospital records at all time points. Linear regression was used to impute SCAS and VAS whilst predictive mean matching was used to impute SF-36-PFS, Chalder Fatigue score, EQ-5D-Y and questionnaire costs due to non-normality of the data.
