# Supplementary figures and images for: Clinical and cost-effectiveness of the Lightning Process in addition to specialist medical care for paediatric chronic fatigue syndrome: randomised controlled trial
Source: Arch Dis Child. 2017 Sep 20;103(2):155–64. doi: 10.1136/archdischild-2017-313375 (PMC5865512; doi:10.1136/archdischild-2017-313375)

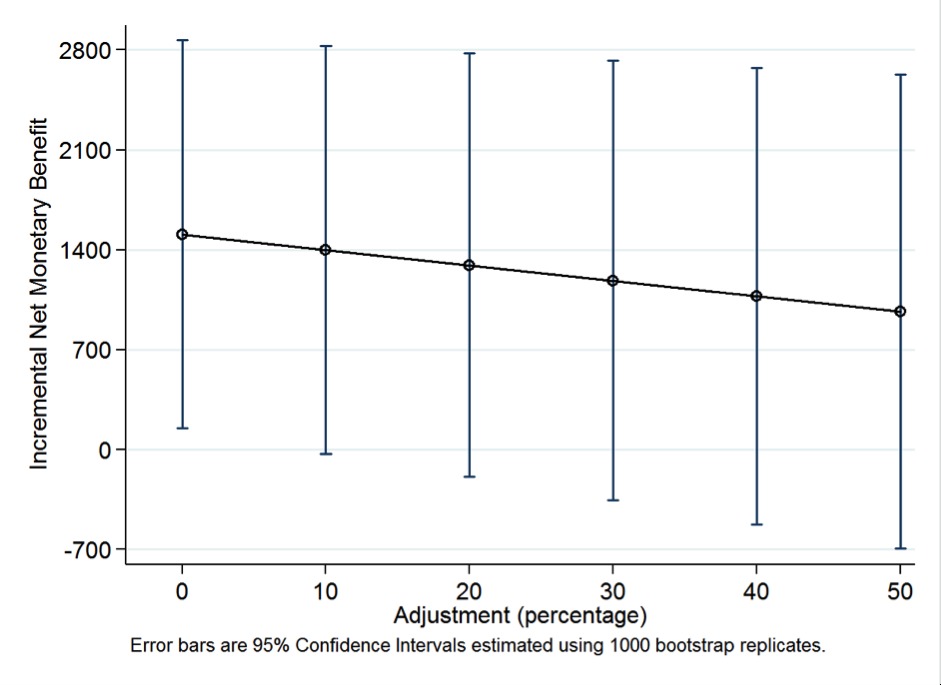

Supplement: Supplementary file 2 [file archdischild-2017-313375supp002.jpg]
