# Supplementary material for: Notice of correction and clarification: Clinical and cost-effectiveness of the Lightning Process in addition to specialist medical care for paediatric chronic fatigue syndrome: randomised controlled trial
Source: Arch Dis Child. 2019 Jul 11;104(10):e4. doi: 10.1136/archdischild-2017-313375corr1 (PMC6889741; doi:10.1136/archdischild-2017-313375corr1)
Supplement: Supplementary data [file archdischild-2017-313375corr1supp001.pdf]

SUPPLEMENTARY FILE: COMPARISON OF FILE ACCEPTED 28 JULY 2017 AND FILE ACCEPTED 12 MARCH 2019

NOTE: Changes marked are the changes made to correct and clarify the article. The file used to generate this comparison was the accepted Word document which may differ slightly from the published version as this is the pre-copy editing file.

Clinical and cost-effectiveness of the Lightning Process in addition to Specialist Medical care for pediatric Chronic Fatigue Syndrome: randomized controlled trial.

Esther Crawley[1] (PhD), Daisy M Gaunt[2 3] (MSc), Kirsty Garfield[2 3](MSc), William Hollingworth[2](PhD), Jonathan AC Sterne[2](PhD), Lucy Beasant[1](MSc), Simon M Collin[1](PhD), Nicola Mills[2] (PhD) and Alan A Montgomery[3 4](PhD)

[1] Centre for Child and Adolescent Health, School of Social and Community Medicine, University of Bristol, Oakfield House, Oakfield Grove, Bristol BS8 2BN

[2] School of Social and Community Medicine, University of Bristol, Canynge Hall, 39 Whatley Road, Bristol BS8 2PS

[3] Bristol Randomised Trials Collaboration, School of Social and Community Medicine, University of Bristol, Canynge Hall, 39 Whatley Road, Bristol, BS8 2PS

[4] Nottingham Clinical Trials Unit, School of Medicine, University of Nottingham, Queen's Medical Centre, Nottingham, NG7 2UH

Corresponding author: Esther Crawley. Centre for Child and Adolescent Health, School for Social and Community Medicine, Oakfield House, Oakfield Grove, Bristol BS8 2BN Email: esther.crawley@bristol.ac.uk. Telephone 0117 331 4099.

ABSTRACT:

Objective: Investigate the effectiveness and cost-effectiveness of the Lightning Process in addition to Specialist Medical Care (SMC) compared to SMC alone, for children with CFS/ME.

Design: Pragmatic randomized controlled open trial. Participants were randomly assigned to Specialist Medical Care (SMC) or SMC plus the Lightning Process (SMC+LP). Randomisation was minimised by age and gender.

Setting: Specialist paediatric CFS/ME service.

Patients: 12-18 year olds with mild/moderate CFS/ME

Main outcome measures: The primary outcome was the SF-36 physical function subscale (PFS) at six months. Secondary outcomes included the SF-36-PFS at three and 12 months, and pain, anxiety, depression, school attendance and cost-effectiveness from a health service perspective at three, six and 12 months.

Results: We recruited 100 participants, of whom 51 were randomized to SMC+LP. between September 2010 and September 2013. We tested the feasibility of running the trial with a feasibility phase (29th September 2010 to 18th September 2012). The full trial was registered in June 2012 when we had determined it was a feasible study. Of the 100 participants, 51 were randomized to SMC+LP. Data from 81 participants were analysed at 6 months. Physical function (SF-36-PFS) was better in those allocated SMC+LP (adjusted difference in means 12.5 [95% CI 4.5, 20.5], p=0.003) and this improved further at 12 months (15.1, [5.8, 24.4], p=0.002). At 6 months, fatigue and anxiety were reduced and at 12 months, fatigue, anxiety, depression and school attendance had improved in the SMC+LP arm. Results were similar following multiple imputation. SMC+LP was probably more cost-effective in the multiple imputation dataset (difference in means in net monetary benefit at 12 months £1474, [95% CI £111 to £2836], p=0.03403) but not for complete cases. This trial is registered with Current Controlled Trials (ISRCTN (http://www.controlled-trials.com/ISRCTN81456207).

Conclusion: The Lightning Process is effective and is probably cost-effective when provided in addition to specialist medical care for mild/moderately affected adolescents with CFS/ME.

INTRODUCTION

Paediatric chronic fatigue syndrome (CFS) or myalgic encephalitis (ME) affectseffects 0.57-2.4%[1-4]of children and is disabling with important impacts on mood[5 6]school attendance[4 7 8] quality of life[9] and family functioning[10]. It is defined as generalised fatigue causing disruption of daily life, persisting after routine tests and investigations have failed to identify an obvious underlying cause[11]. A minimum of three months of fatigue is required before the diagnosis can be made[12]. On average, those affected miss a year of school overall and half are bedbound at some stage [13 14].

There is a limited evidence base for treatment of paediatric CFS/ME[12 15 16]. Three randomized trials have shown that Cognitive Behavioural Therapy (CBT) delivered individually[17][17], with biofeedback[18][18] or via the internet[19] is effective at six months compared to waiting list or usual medical care. All three studies reported improvements in fatigue, school attendance and a reduction in disability. Family-focused CBT appears to be as effective as psycho-education in terms of school attendance at six months and recovery at 24 months[20 21]. However, even with effective treatment, over a third of children[19 20] have not recovered at six months and 12 months[22][22] and 21%[21] to 36%[22] are still unwell (for example attending school less than 70% of the time) at 24 months. There is therefore an urgent need to find more effective treatments.

The Lightning Process® (LP) is developed from osteopathy, life coaching and neuro-linguistic programming and is used for a variety of conditions including CFS/ME. Clients read information, attend three group sessions and then receive follow-up phone calls[23] .[23]. More than 250 children use LP for their CFS/ME each year in the UK (at a cost of ~£620 each), but there are no reported studies investigating its effectiveness, cost-effectiveness or side effects. LP is not available in the NHS. Having shown that recruitment, randomisation and data collection was feasible and acceptable[24][24] we conducted a randomized trial to investigate the effectiveness and cost-effectiveness of LP in addition to Specialist Medical Care (SMC), compared to SMC alone, for children with CFS/ME.

METHODS

Study design and participants

A detailed description of the study protocol has been reported[25]. Between September 2010 and April 2013 we recruited participants after clinical assessment by the Bath/Bristol paediatric CFS/ME service, a large regional and national NHS specialist service.[25]. Between September 2010 and September 2013 we recruited participants after clinical assessment by the Bath/Bristol paediatric CFS/ME service, a large regional and national NHS specialist service. We tested the feasibility of running this trial with a feasibility phase (29th September 2010 to 18th September 2012). We determined the trial was feasible in June 2012 and registered the full trial (31st July 2012). We applied for an amendment to recruit children into the full trial as opposed to a feasibility trial (see web table 1 for detailed description of amendments). Full trial first randomisation was the 19th September 2012. We continued seamlessly with participant recruitment without any interim between-group comparison of participant outcome data from the feasibility phase.. Children from both phases (feasibility and full) were analysed. Children were diagnosed with CFS/ME after a thorough assessment which included screening for other disorders associated with fatigue[12]. Baseline data were collected at this assessment. Children were eligible if they had CFS/ME, were aged 12-18, spoke English and were not housebound.

Randomisation and masking

Allocation to trial arms was in equal proportions using minimisation by age (12-15/16-18 years) and gender, weighted towards minimising the imbalance in trial arms with probability 0.8. Allocation was concealed using a telephone-based interactive voice response system, created and maintained by the Bristol Randomised Trials Collaboration, and accessed by the recruiting researcher. This was an open study: the randomized intervention was conveyed after obtaining consent, during the

recruitment interview so that participants, parents, therapists and researchers were aware of treatment allocation. Data analyses were conducted using masked treatment codes.

Interventions

All participants were offered Specialist Medical Care (SMC)[12] which focused on improving sleep and using activity management to establish a baseline level of activity (school, exercise and social activity) which is then gradually increased. Sessions were delivered by a range of trained and supervised professionals including doctors, psychologists, physiotherapists and occupational therapists in family-based rehabilitation consultations. Follow-up sessions were either face to face or by telephone. The number and timing of the sessions were agreed with the family depending on each adolescent's needs and goals. Those with significant anxiety or low mood were offered additional CBT. Participants could choose to use physiotherapist-delivered Graded Exercise Therapy, which provides detailed advice about exercise and focuses on an exercise programme rather than other activities.

Participants randomized to SMC+LP were asked to read information about LP and complete an assessment form with their parents to identify their goals and describe what they had learnt. They then had a telephone call with an LP practitioner (Appendix 1) to discuss attending an LP course consisting of three four-hour sessions on consecutive days run with groups of two to five young people. Each had a theory session with taught elements on the stress response, how the mind and body interact, and how thought processes can be either helpful or negative. This was followed by group discussion where the language used was discussed and in some cases challenged, and where participants were encouraged to think about what they could take responsibility for and change. In the practical session, participants identified a goal they wished to achieve (such as standing for longer) and were given different cognitive (thinking) strategies before and whilst the goal was attempted. They were also asked to identify a goal to attempt at home. After the course, young people were offered at least two follow-up phone calls with a LP Practitioner.

Lightning Process practitioners have completed a Diploma through the Phil Parker Training Institute in Neurolinguistic Programming, Life Coaching and Clinical Hypnotherapy. This diploma is examined through written and practical exams and is accredited by the British Institute of Hypnotherapy and NLP. Following the Diploma, Lightning Process practitioners undertake a further course to learn the tools and delivery required for the Lightning Process after which they must pass both a practical and written exam. Practitioners undertake supervision and CPD in order to further develop their skills and knowledge. They are regulated by the Register of Lightning Process practitioners, adhere to a Code of Conduct, and there is a Professional Conduct Committee that oversees complaints and professional practice issues.

Outcomes

The primary outcome was the SF-36 physical function subscale (SF-36-PFS[26]) analysed as a continuous variable collected at six months post-randomisation. We chose the SF-36 based on qualitative work conducted in the feasibility phase of the study.[24] We have reported that parents and participants "commented that the school attendance primary outcome did not accurately reflect what they were able to do, particularly if they were recruited during, or had transitioned to, A levels during the study". In addition, "we were aware of some participants who had chosen not to increase school attendance despite increased activity." We therefore concluded that: "trials involving 17 and 18 year olds should consider alternative primary outcome measures to school attendance as it is difficult to assess for those transitioning from GCSEs to A levels, and may not be appropriate for those who do not consider school attendance their primary goal". At this stage, our recommendation was that a "full study uses other primary outcomes, such as the SF-36 or the Chalder Fatigue Scale and uses school attendance as a secondary outcome." These findings informed

our application for our ethical amendment to a full study in 2011 (see Web table 1). And were published in our feasibility paper in 2013.[24]

Qualitative interviews with SMILE participants then formed part of a larger study which described the conceptual model for paediatric CFS/ME.[27] In this study, physical activity (or disability) is described by children as being pivotal because of the impacts on social participation and emotional wellbeing. Whilst school was deemed to be an important contextual factor, these qualitative results led us to choose the SF-36-PFS as a primary outcome with school attendance as a secondary outcome. There was no analysis of any outcome data during or after the feasibility phase until the entire trial was completed.

Secondary outcomes were the SF-36-PFS at three and 12 months, and school attendance (days per week), the Chalder Fatigue scale[27][28], pain (visual analogue scale), Hospital Anxiety and Depression Scale (HADS)[29], Spence Children's Anxiety Scale (SCAS)[30] and quality-adjusted life years (QALYs, derived from the EQ-5D-Y)[28] at three, six and 12 months. Pain was measured by a visual analogue scale at six months. All were self-completed by participants. Participants also completed the Hospital Anxiety and Depression Scale (HADS)[29] and the Spence Children's Anxiety Scale (SCAS)[30] at assessment,[31] at three, six and 12 months. At three, six and 12 months parents completed an adapted 4 item Work Productivity and Activity Impairment: General Health V2.0 (WPAI:GH) questionnaire (V2.0)[31][32] and a resource use questionnaire assessing their child's health service use (e.g. GP or specialist care), educational service use (e.g. school counsellor), health related travel and other family costs.

Time windows for questionnaire return were pre-specified as six weeks after the three month follow-up, six weeks before or up to three months after the six month follow-up, and three months before or after the 12 month follow-up. Those who had not responded within one week were sent a reminder letter with a reduced set of questionnaires (SF-36-PFS, Chalder Fatigue scale and school

attendance). From February 2011 non-responders were telephoned by a researcher and the SF-36-PFS and Chalder Fatigue scale were completed over the phone to improve follow up rates.

Sample size

~~We used a~~A consensus definition for a small clinically important difference ~~of 10 points~~ on the SF-36-PFS at six months follow up is 10 points~~[32]~~[33]~~. 32~~. However, we did not want to ~~50 participants in each arm are required to~~miss a smaller but still potentially important effect of as low as 8 points. To detect a between-group difference of 8 to 10 points ~~on the SF-36-PFS (SD 10) at six months~~ with 90% power~~ and~~, 1% two-sided significance~~.~~ and standard deviation of 10 requires between 32 and 50 participants per group for analysis. Allowing for 10 to 20% non-collection of primary outcome data, we aimed to recruit between 80 ~~to~~(32*2/0.8) and 112 (50*2/0.9) participants.

Statistical analysis

The statistical analysis plan was agreed by the study management group and published on our website prior to analyses. The primary analysis compared mean SF-36-PFS scores at six months according to randomized allocation among participants with measured outcomes, using multivariable linear regression adjusting for baseline values of the outcome, baseline age and gender. Similar regression analyses were conducted for secondary outcomes. Sensitivity analyses of the primary outcome adjusted for variables for which there was baseline imbalance; excluded those recruited up to 31 January 2011 preceding the protocol amendment to allow collection of follow up data by phone; and used multiple imputation of missing data~~ (see Web Appendix 1 for details)~~. Missing items in partially completed scales (Chalder Fatigue and SF-36-PFS) or subscales (SCAS and HADS anxiety and depression) were imputed using the mean of completed items, if only one item (or two for the SCAS subscales) was missing. If more items were missing the whole scale or subscale was scored as missing. ~~Twelve month outcome data were analysed similarly.~~ We conducted a repeated measures analysis using all follow-up SF-36-PFS scores, with and without an interaction between

allocation arm and time, to investigate whether between-group differences remained constant over time. We did not analyse three month outcomes except in this repeated measures analysis for SF-36-PFS as these were unlikely to be informative since the primary follow up was at six months. We estimated the Complier Average Causal Effect (CACE), using instrumental-variables linear regression estimated via the generalized method of moments (GMM), of LP among compliers, defined as participants in the SMC+LP arm who completed all of the LP course.

Pre-specified subgroup analyses explored differences in treatment effect according to baseline age (<15 versus 15 to 17), gender, severity (none versus some school attendance at baseline) and co-morbid anxiety (> or ≤12 on the HADS anxiety subscale) for the primary outcome, by adding an interaction term to the primary analysis multivariable linear regression model.

Health economic analyses

We conducted a cost-utility analysis of SMC+LP from the health service and public sector perspective. We estimated the incremental net monetary benefit (iNMB) of SMC+LP versus SMC, at a threshold willingness-to-pay of £20,000 (~US$30,000) per QALY[33] .[34]. In the primary analysis, we used the cost of LP charged to the trial (mean £567). In sensitivity analyses we: (1) used the price of LP outside of trial (£620; July 2014 price); (2) estimated cost of providing the LP intervention within the UK health service (Web Table 12). SMC outpatient attendances were extracted from hospital records. Other healthcare use was based on parent-report. Resource use was combined with 2013 unit costs (Web Table 12)[34-37][35-38]. In the absence of a paediatric valuation for the EQ-5D-Y, we used the UK adult tariff[38]. QALYs were estimated using the area under the curve[39] .[39]. QALYs were estimated using the area under the curve[40]. Incremental costs, QALYs and net benefits were adjusted for baseline values, age, gender and for variables where there was baseline imbalance. Non-parametric bootstrapping methods were used to calculate normally distributed 95% confidence intervals around the iNMB. The probability that SMC+LP is cost-effective at varying willingness-to-pay thresholds was estimated using a cost-effectiveness acceptability curve. Where one item of the

EQ-5D-Y was missing (n=3), the mean of the other domains (rounded to the nearest integer) replaced the missing value. A high proportion of participants had missing resource use data at three, six, and 12 months. Therefore we conducted two analyses based on the complete case and multiply imputed datasets. (Web appendix 2).

All analyses were conducted using Stata (StataCorp. 2013. Stata Statistical Software: Release 13.1. College Station, TX: StataCorp LP).

Ethical review

A favourable ethical opinion was given on 8 September 2010 (reference 10/H0206/32) by South West 2 Local Research Ethics Committee. Two favourable opinions were provided for amendments to study documents and protocol on 31 May 2011 and 6 September 2012.

RESULTS

Of 657 children assessed in the specialist CFS/ME clinic during the recruitment period, 631 were assessed for study eligibility and 310 were eligible (Figure 1). Among those eligible 136 consented to receiving further information and 100 were randomized: 49 to SMC only and 51 to SMC+LP. Recruitment was stopped after the 100th participant was randomized. 56 of these participants were included in the report of whether it was feasible to conduct this RCT.[24] Recruitment was stopped after the 100th participant was randomized. Eligible children and adolescents who found out more about the trial but were not randomized had lower anxiety and depression scores and attended more school (Web Table 23). Participants' mean age was 14 years, 76 were female and all described themselves as British. Participants were disabled by their fatigue: only seven were attending full time school and 47 described themselves as attending two days or less school a week.

Participants' characteristics at baseline were balanced between arms except for pain and anxiety (SCAS) scores (Table 1), which was adjusted for in sensitivity analyses. The imbalance in pain and SCAS scores were in opposite directions suggesting that the two arms were not systematically different. Five participants withdrew from the study: two from the SMC and three from the SMC+LP arm. Outcome data were collected from 92 participants on at least one follow-up occasion. Baseline characteristics were similar between those who did (n=82) and did not (n=18) provide primary outcome data at six months (Web Table 34). The mean (SD) time between clinical assessment and primary outcome collection was 6.8 (1.0) and 6.8 (0.7) months in the SMC and SMC + LP arms respectively. Treatment as allocated was received by 46 (94%) and 39 (76%) participants in the SMC and SMC+LP arms respectively. Three participants (3/39, 8%) in the SMC+LP arm received the LP course after completing the 6 month follow-up, these participants were included in the analyses.

Mean SF-36 physical function improved more over time in participants allocated to SMC+LP than in those allocated to SMC (Figure 2). Participants allocated to SMC+ LP had better physical function at six months than those allocated to SMC (Table 2, adjusted difference in means 12.5 [95% CI 4.5,

20.5], p=0.003). This difference increased to 15.1 (95% CI 5.8, 24.4, p=0.002) at 12 months. These differences were similar when additionally adjusted for baseline anxiety (SCAS) and pain (VAS), when analyses were restricted to participants recruited from February 2011, and with multiple imputation of missing data (Table 2). The average between-arm difference in physical function across both 6 and 12 month follow-up was 14.4 (95% CI 7.3, 21.5),, p<0·001. The). When compliance was taken into account using CACE analyses, the estimated effect of LP (using CACE analyses) among compliers at 6 and 12 months was increased compared with the ITT estimate (Table 2).).. There was little evidence that the effect of LP+SMC compared with SMC on the primary outcome differed according to baseline age, anxiety or school attendance (all interaction p values >0.3). There was weak evidence (Web Table 45) that the effect in males (adjusted difference in means 26.6, [95%CI 8.9, 44.3]) was greater than that in females (adjusted difference in means 9.0, [95% CI 0.2, 17.8]) with an interaction p-value of 0.08.

Participants in the SMC+LP arm had less fatigue (adjusted difference in means -4.7 [95% CI -7.9, -1.6], p=0.003) (Table 3) than those allocated to SMC and a greater improvement in anxiety symptoms measured by both the HADS (-3.3, [95% CI -5.6, -1.0], p=0.005) and the SCAS (-8.7, [95% CI -16.9, -0.5], p=0.03904) scores at six months. The difference in means in fatigue score and HADS anxiety score were somewhat smaller at 12 months (-3.2 [95% CI -6·3, -0·1] and -2·8 [-4·7, -0·8] respectively). However the difference in means in SCAS anxiety was greater at 12 months (-12·1 [95% CI -20·1, -4·1] and there was evidence that there was less depression among participants allocated to SMC+LP than those allocated to SMC at 12 months (adjusted difference in means in HADS depression score -1.7 [95% CI -3·3, -0·2] p=0.03003). Participants allocated to SMC+LP had better school attendance at 12 months than those allocated to SMC (adjusted difference in means 0.9 days of school per week [95% CI 0.2, 1.6] p=0.018). Pain02). Mean pain scores were lower in participants allocated to SMC+LP compared with those allocated to SMC at both six and 12 months, but confidence intervals were wide.

Five adverse events were reported (three in the SMC+LP arm). Four were related to participants and one to a parent. None were attributed to either SMC or LP. Physical function at six months deteriorated in nine participants, of whom eight were in the SMC arm. Five of the nine had deterioration of ≤ 10 on the SF-36 physical function subscale (range 0-100) which is less than the Minimal Clinically Important Difference (MCID).

EQ-5D-Y questionnaires were completed by 65, 82 and 80 participants at three, six and 12 months respectively (Figure 3); 56 completed EQ-5D-Y at all three follow-up time points. EQ-5D-Y scores were generally higher in the SMC+LP group. Differences in quality-adjusted life year (QALYs) were evident at 12 months in the multiple imputation dataset (Table 4, adjusted difference in means 0.095 QALYs, [95% CI 0.030 to 0.160], p=0.004), but in the complete case dataset the confidence interval included zero (adjusted difference in means 0.080 QALYs, [95% CI -0.064 to 0.225], p=0.2763).

Complete healthcare use questionnaires were returned by between 55 (55% at 12 months) and 56 (56%56 participants at 3 and 6 months) participants and 55 at 12 months, but only 30 (30%)participants completed these questionnaires at all three time points (see web table 5 for details).. The initial cost of LP was not fully offset by marginally lower costs of other care over the 12 month period. The incremental cost (Table 4) of SMC+LP was higher in both complete case (difference in means £445, [95% CI -57 to 947], p=0.08208) and multiple imputation datasets (difference in means £390, [95% CI 189 to 591], p=<0.000005).

Table 4 shows that in the multiple imputation dataset there was good evidence that SMC+LP was more cost-effective than SMC alone (incremental net monetary benefit (iNMB) £1508, [95% CI £148 to £2869], p=0.03403), although the evidence was much weaker in the complete case dataset (Figure 4, web table 6). Sensitivity analyses varying the unit cost of LP treatment made no difference to this conclusion (Web Table 6, Web figure 17). Sensitivity analyses assuming costs and QALYs are

not missing at random [40] did not alterreduced the conclusionstrength of the evidence that SMC+LP was likely to be cost-effective, but reduceddid not alter the strength of the evidenceconclusion.

DISCUSSION

This is the first randomized trial investigating the effectiveness of the Lightning Process for any condition. It is the first trial that has demonstrated the effectiveness of an intervention other than CBT for paediatric CFS/ME. Addition of the Lightning Process to Specialist Medical Care improved physical function at 6 months in adolescents with CFS/ME and this difference increased at 12 months. The addition of LP also improved fatigue and anxiety at six months and fatigue, anxiety and depression at 12 months. Participants in the LP arm were attending one day more of school a week at 12 months on average. The initial cost of LP was not fully offset by lower subsequent costs of health care, but the improvements in health-related quality of life meant that SMC+LP is probably cost-effective using a threshold for a quality-adjusted life years of £20,000 (~US $30,000). Participants in the SMILE trial did not have any serious adverse events attributable to either treatment arm. The majority of those who experienced a deterioration in physical function had a deterioration of ≤ 10 on the SF-36 physical function subscale. The lack of serious adverse events is consistent with other treatment trials in CFS/ME[4041].

Strengths of the study include its randomized design and that we followed patientsfollow up for 12 months. Participants received specialist medical care that is currently being delivered in the UK Health Service by a multidisciplinary team, and the Lightning Process as it is currently provided. More participants were lost to follow-up in the SMC arm, but baseline characteristics were similar in those followed and not followed up. Complete healthcare use questionnaires were returned by only 55 or 56 participants at each time point. We used multiple imputation to correct for potential bias due to missing data and conducted sensitivity analyses restricted to participants recruited after the protocol changed to collect primary outcome data by telephone, which improved follow-up rates suggesting results were robust. We pre-defined the clinically important difference (10 points) on the SF-36-PFS and the difference in means was greater than this at both 6 and 12 months. The study was not blinded, so that patient-reported outcomes may have been affected by participants' knowledge

of the group to which they were randomized. Only 36 (70%) of those allocated LP attended the full course prior to the 6 month follow-up but we estimated the effect in all those who completed the full LP course. The study was originally planned as a feasibility study and although randomised, was not registered on a trial registry, since the aim was to investigate feasibility rather than effectiveness of the intervention. After establishing feasibility, we applied to register the full trial in June 2012. At this time, the results of our feasibility work suggested we could use either SF-36-PFS or Chalder Fatigue scale or both which we registered as primary outcomes. We decided to use just the SF-36-PFS and published this in 2013 and in our analyses plan. We did not update the ISTCRN site until 2018, however, we uploaded the relevant publications in 2016 and the study website had the updated analyses plan.

The Lightning Process may not be suitable for all children and adolescents. Fewer than 30% of eligible children were randomized. We do not know why the majority did not want to take part in the trial but it may be because they did not want to take part in groups or travel for three consecutive days. We felt it would be unethical to have a control group without treatment and therefore we only know that LP is effective in addition to specialist medical care and not whether it is effective on its own. We only recruited children aged 12 and over who were not housebound and who spoke English. We do not know whether LP is effective, acceptable or feasible for those who are severely affected, less than 12 years old or do not speak English., or do not speak English. The study was registered after demonstrating feasibility. The analysis includes participants who were recruited prior to registration of the study. This does not comply with ICMJE and BMJ guidance on trial registration. The reasons for this have been explained in the paper.

Participants in both treatment arms improved. Those receiving SMC alone had a mean improvement that was similar to that seen in adults receiving CBT or GET[4041]. The improvement in SF-36-PFS in those receiving SMC+LP is consistent with those receiving treatment in previous paediatric trials

investigating both family based and individual CBT[17 20]. We pre-defined the minimally clinically important difference as 10 points on the SF-36-PFS based on consensus statements. Subsequent work by our team has shown that this is a clinically significant change in physical function for children with CFS/ME.[42] Ten points equates to a minimum of two step changes on the SF-36-PFS. This can be either one step change on two questions, or two step changes on one question. The SF-36 asks: "Does your health limit you in these activities? If so, how much? As an example, one step change could be: "Yes, limited a lot" to "Yes, limited a little" or "Yes, limited a little" to "No, not limited at all" to different questions such as "climbing several flights of stairs" or "walking 100 yards" or "walking half a mile". The participants in our study who received SMC only, did not improve as much as other trials investigating CBT[17 20] which may be because on average they had less than half the number of treatment sessions. As we did not compare LP with either a full course of only CBT or GET we do not know if LP is more of less effective than either of these treatment approaches.

Participants in the SMC+LP arm maintained or increased improvements compared to SMC alone at 12 months and this was true for both the Intention to Treat (ITT) and the Complier Average Causal Effect (CACE) analyses. This is in contrast to previous trials investigating internet based CBT where the treatment effects were sustained but the difference between the two trial arms was reduced at 12 months compared to three months[19 22]and family focused CBT versus psycho-education where treatment differences at three months were not maintained at six or 12 months[20].

There is only one study[23] investigating LP which used qualitative interviews to explore the views of nine 14-26 year olds about their experiences. The main difference between LP and CBT appears to be the emphasis placed on physiological responses and causal attributions[23] but we do not know whether these explain the greater effectiveness of LP. We do not know which aspects of the LP are the most important or helpful. Some young people who received LP value the theory, others the practical sessions or the homework[23]. Further research is needed to understand why LP improves outcomes at six and 12 months and which aspects of the LP contribute to its effectiveness.

There is only one study[23] investigating LP which used qualitative interviews to explore the views of nine 14-26 year olds about their experiences. The main difference between LP and CBT appears to be the emphasis placed on physiological responses and causal attributions[23] but we do not know whether these explain the greater effectiveness of LP. We do not know which aspects of the LP are the most important or helpful. Some young people who received LP value the theory, others the practical sessions or the homework[23]. Further research is needed to understand why LP improves outcomes at six and 12 months and which aspects of the LP contribute to its effectiveness.

Conclusions

The addition of the Lightning Process to Specialist Medical Care may be helpful to children with CFS/ME. However, this study needs to be replicated before the Lightning Process should be offered in the NHS.

Contributors

EC obtained the funding, designed and supervised the trial. DG conducted the statistical analysis and compiled the results tables. KG conducted the health economic analyses and compiled the health economic tables. WH contributed to the study design and supervised the health economic analyses. JS contributed to the study design and co-supervised the statistical analyses. LB contributed to the study design and running the trial. SC managed the data and contributed to the statistical analyses. NM contributed to the study design. AM contributed to the study design and co-supervised the statistical analyses. All authors contributed to the data interpretation and writing the paper. EC is the guarantor.

Competing interests

All authors have completed the ICMJE uniform disclosure form at www.icmje.org/coi_disclosure.pdf and declare: Financial support for the submitted work was received from The Linbury Trust and The Ashden Trust; EC and SC have received fellowship grants from The NIHR; JACS has received grants from the NIHR; EC runs the specialist CFS/ME service at Royal United Hospital NHS Foundation Trust, has received one grant from MRC, one grant from the NIHR and is medical advisor to the Sussex & Kent ME/CFS Society.

The authors declare they did not receive any funding from the Lightning process.

Funding Support

This trial was funded by the Linbury Trust (grant number LIN2038) and the Ashden Trusttrust (grant numbers ASH1062, 1063, 1064). No member of the Linbury Trust or the Ashden Trust had any involvement in the design, analyses or in writing the paper. Dr Crawley was funded by an NIHR Clinician Scientist fellowship followed by an NIHR Senior Research Fellowship (SRF-2013-06-013) during the trial. SC was funded by an NIHR Post Doctoral Fellowship during the analyses of the trial. This report is independent research and the views expressed in this publication of are those of the authors and not necessarily those of the NHS, the National Institute for Health Research of the Department of Health.

Role of the funding source and sponsor

The funders and the sponsor of the study had no role in the design and conduct of the study; collection, management, analysis, and interpretation of the data; preparation, review, or approval of the manuscript; and decision to submit the manuscript for publication. All researchers involved in this study were independent from both the funders and the sponsor.

Data Access, Responsibility, and Analysis

The authors had access to all the data. The corresponding author had full access to all the data in the study and had final responsibility for the decision to submit for publication.

Acknowledgements

We are grateful to all the children and families who took part in this study and to the Bath specialist CFS/ME service who recruited them. We thank Kate White, Anu Ganupathy and Debbie Johnson who helped with data collection and recruitment during the study. We are grateful to Phil Parker and the LP central office who provided advice and information on costs. No member of the LP team had any involvement in the analyses or in writing the paper.

Transparency declaration

The lead author (and guarantor) affirms that this manuscript is an honest, accurate, and transparent account of the study being reported; that no important aspects of the study have been omitted; and that any discrepancies from the study as planned have been explained.

Data sharing Statement

Data sharing: for information on how to access data, please check

DOI 10.5523/bris.1myzti8qnv48g2sxtx6h5nice7

The authors had access to all the data. The corresponding author had full access to all the data in the study and had final responsibility for the decision to submit for publication. Given the nature of this dataset, access is controlled. Requests are referred to the University of Bristol Data Access Committee for approval before data can be released under an appropriate data access agreement. For details on how to access data, see the repository record at http://dx.doi.org/10.5523/bris.1myzti8qnv48g2sxtx6h5nice7

Table 1. Characteristics of the randomized participants at baseline

	SMC group	N	SMC ~~plus~~+ LP group	N
**Demographic characteristics**				
Mean age (SD)	14·5 (1·6)	49	14·7 (1·4)	51
Number female (%)	38 (~~77·6~~78)	49	38 (~~74·5~~75)	51
Median months from onset of illness to baseline assessment (25th percentile, 75th percentile)	12 (7·0, 22·0)	49	12 (8·0, 18·0)	49
**Clinical characteristics**				
Mean SF-36 physical function score[1] (SD)	56·0 (21·5)	49	53·0 (18·8)	50
Mean Chalder Fatigue score[2] (SD)	25·1 (4·2)	49	25·0 (4·2)	50
Mean pain VAS[2] (SD)	42·4 (29·4)	48	51·6 (28·5)	48
Mean SCAS[2] (SD)	40·3 (20·1)	48	29·8 (16·9)	49
Mean HADS Anxiety score[2] (SD)	10·4 (4·4)	48	8·8 (4·5)	51
Mean HADS Depression score[2] (SD)	8·1(4·4)	48	7·5 (3·1)	50
Mean EQ-5D score[1] (SD)	0·34 (0·36)	49	0·31 (0·34)	51
School attendance in the previous week N (%):				
None	7 (14~~·3~~%)	49	6 (12~~·0~~%)	50
0·5 day	7 (14~~·3~~%)	49	5 (10~~·0~~%)	50
1 day	3 (6~~·1~~%)	49	3 (6~~·0~~%)	50
2 days	8 (16~~·3~~%)	49	8 (16~~·0~~%)	50
3 days	12 (24~~·5~~%)	49	12 (24~~·0~~%)	50
4 days	9 (18~~·4~~%)	49	12 (24~~·0~~%)	50
5 days	3 (6~~·1~~%)	49	4 (8~~·0~~%)	50

HADS: Hospital Anxiety and Depression Scale~~; IQR: Interquartile range~~; SCAS: Spence Children's Anxiety Scale; SD: Standard deviation; SF-36: The 36-item short-form health survey; VAS: Visual Analogue Scale. All results rounded to 1 d.p. or whole percentage points [1]Higher score=fewer symptoms, better function. [2]Higher score=more symptoms, poorer function.

Table 2: Primary outcome

SF-36 physical function	SMC group		SMC plus+ LP group		Crude difference in means (95% CI), P value	Adjusted difference in means [2] (95% CI), P value	N	Adjusted difference in means [3] (95% CI), P value	N
	Mean	N	Mean	N					
**Baseline**	56·0	49	53·0	50					
**6 months (primary outcome)[1]**	70·2	37	81·7	45	11·5 (3·1, 19·8), 0·008	12·5 (4·5, 20·5), 0·003	81	12·9 (3·6, 22·1), 0·007	76
Children recruited from 1 Feb 2011	70·5	34	81·4	39	10·9 (1·8, 20·0), 0·020	11·8 (3·2, 20·3), 0·008	72	13·1 (3·3, 22·8), 0·009	68
With imputation of missing data	70·9	49	81·1	51	10·2 (2·2, 18·2), 0·013	11·3 (3·8, 18·9), 0·004	100	11·8 (3·6, 19·9), 0·005	100
Effect among compliers (CACE)					15·2 (5·0, 25·3), 0·003	16·6 (6·9, 26·2), 0·001	81	17·5 (7·1, 28·0), 0·001	76
**12 months[1]**	71·8	38	86·1	42	14·2 (4·6, 23·8), 0·004	15·1 (5·8, 24·4), 0·002	79	16·4 (6·1, 26·8), 0·002	73
With imputation of missing data	73·1	49	85·5	51	12·4 (3·3,21·5), 0·008	12·6 (4·0, 21·3), 0·005	100	14·7 (5·6,23·9), 0·002	100
Effect among compliers (CACE)					16·2 (5·6, 26·7), 0·003	17·1 (7·0, 27·3), 0·001	79	18·6 (6·9, 30·4), 0·002	73
**Average of 3, 6 and 12 month differences[4,5]**						13.6 (6.7, 20.4), <0·001	90	13.5 (6.0,21.0), <0·001	84
**Average of 6 and 12 month differences[4,6]**						14.4 (7.3, 21.5), <0·001	87	14.9 (7.0, 22.7), <0·001	81

CACE: Complier Average Causal Effect

[1]Higher score=fewer symptoms, better function. [2]Adjusted for age, gender and baseline outcome. [3]Adjusted for age, gender, baseline outcome, baseline SCAS and VAS. [4]Based on a repeated measures analysis that was additionally adjusted for time-point as a categorical variable. [5]time as categorical by group interaction, adjusted for age, gender and baseline outcome p=0.8. [6]time as categorical by group interaction, adjusted for age, gender and baseline outcome p=0.7

Table 3: Secondary outcomes

	SMC group		SMC plus+ LP group		Crude difference in means (95% CI), P value	Adjusted difference in means[3] (95% CI), P value	N	Adjusted difference in means[4] (95% CI), P value	N
	Mean	N	Mean	N					
Chalder Fatigue score 6 months[1]months[2]	19·8	37	14·4	44	-5·4 (-8·6, -2·1), 0·001	-4·7 (-7·9, -1·6), 0·003	80	-5·4 (-8·9, -1·9), 0·003	76
Chalder Fatigue score 12 months[1]months[2]	15·7	38	12·3	42	-3·4 (-6·6, -0·1), 0.04104	-3·2 (-6·3, -0·1), 0·04505	79	-4·0 (-7·2, -0·7), 0·01702	74
Pain VAS 6 months[1]months[2]	32·8	28	23·4	33	-9·5 (-23·5, 4·6), 0·1832	-11·3 (-23·0, 0·3), 0·05706	58	-9·3 (-21·1, 2·6), 0·1241	58
Pain VAS 12 months[1]months[2]	32·0	27	21·8	32	-10·2 (-24·6, 4·2), 0.1612	-9·4 (-21·5, 2·7), 0·1251	56	-6·5 (-19·4, 6·5), 0·3213	54
SCAS 6 months[1]months[2]	37·4	28	24·7	33	-12·7 (-22·0, -3·3), 0·009	-8·7 (-16·9, -0·5), 0·03904	61	-10·0 (-18·5, -1·5), 0·02202	58
SCAS 12 months[1]months[2]	36·3	27	19·6	31	-16·7 (-25·9, -7·5), 0·001	-12·1 (-20·1, -4·1), 0·004	56	-14·5 (-22·4, -6·7), <0·001	52
HADS Anxiety score 6 months[1]months[2]	9·7	28	6·1	33	-3·7 (-6·0, -1·3), 0·003	-3·3 (-5·6, -1·0), 0·005	60	-3·5 (-5·6, -1·5), 0·001	57
HADS Anxiety score 12 months[1]months[2]	8·3	27	5·3	33	-3·1 (-5·2, -0·9), 0·006	-2·8 (-4·7, -0·8), 0·006	59	-2·6 (-4·7, -0·4), 0·019	53
HADS Depression score 6 months[1]months[2]	5·9	28	4·2	33	-1·7 (-4·0, 0·6), 0·1411	-1·6 (-3·9, 0·7), 0·1612	59	-1·5 (-3·5, 0·5), 0·1291	57
HADS Depression score 12 months[1]months[2]	4·6	27	2·8	33	-1·9 (-3·6, -0·2), 0·03303	-1·7 (-3·3, -0·2), 0·03003	58	-1·8 (-3·4, -0·1), 0·03704	53
School/college attendance in the previous week 6 months[2]months[1] (days)	2·6	37	3·2	41	0·7 (-0·1, 1·4), 0·08308	0·7 (0·0, 1·4), 0·06406	77	0·6 (-0·2, 1·4), 0·1351	72
School/college attendance in the previous week 12 months[2]months[1] (days)	3·1	36	4·1	34	1·0 (0·2, 1·7), 0·01001	0·9 (0·2, 1·6), 0·01802	69	1·0 (0·2, 1·8), 0·01201	65

HADS: Hospital Anxiety and Depression Scale; SCAS: Spence Children's Anxiety Scale; SF-36: The 36-item short-form health survey; VAS: Visual Analogue Scale

[1] Higher score=more symptoms, poorer function. [2]Higher score=fewer symptoms, better function. [2] Higher score=more symptoms, poorer function. [3]Adjusted for age, gender and baseline outcome. [4]Adjusted for age, gender, baseline outcome, baseline SCAS and VAS (as appropriate).

Table 4: MIAnalysis of Multiple Imputation and complete case analysisdata of Total HC + LP costs and QALYs and NMB (£20k) at 6 months and at 12 months; by treatment group, all adjusted for baseline value, age, sex, baseline SCAS and baseline VAS

	SMC			SMC plus+ LP			Incremental difference		
	Mean	(SE)	n	Mean	(SE)	n	(95% CI)		n
**6 Month complete case**									
Total cost (£)	942	(89)	13	1563	(127)	21	621	(323, 919)	34
QALYs	0.252	(0.021)	22	0.259	(0.016)	32	0.008	(-0.057, 0.073)	34
NMB at £20,000 per QALY	4225	(578)	13	3762	(461)	21	-464	(-1852, 925)	34
**6 Month Imputed**									
Total cost (£)	1123	(66)	49	1517	(54)	51	394	(236, 553)	100
QALYs	0.247	(0.015)	49	0.274	(0.014)	51	0.026	(-0.015, 0.068)	100
NMB at £20,000 per QALY	3819	(328)	49	3954	(276)	51	135	(-733, 1003)	100
**12 Month complete case**									
Total cost (£)	1369	(160)	11	1814	(211)	16	445	(-57, 947)	27
QALYs	0.551	(0.039)	21	0.597	(0.032)	30	0.080	(-0.064, 0.225)	27
NMB at £20,000 per QALY	9454	(1202)	11	10615	(1113)	16	1161	(-1966, 4289)	27
**12 Month imputed**									
Total cost (£)	1612	(84)	49	2002	(67)	51	390	(189, 591)	100
QALYs	0.533	(0.025)	49	0.628	(0.021)	51	0.095	(0.030, 0.160)	100
NMB at £20,000 per QALY	9042	(521)	49	10551	(427)	51	1508	(148, 2869)	100

QALY: quality-adjusted life years; NMB: net monetary benefit. For the complete case results, the N in the final column may not equal the sum of the Ns in the preceding columns. The Ns in the preceding columns include participants with complete cost **or** QALY data, and the N in the final column only includes participants with complete cost **and** QALY data

What is already known on this topic

Paediatric CFS/ME is relatively common with a negative impact on school, mood and quality of life

Even with effective treatment, a significant number of children have not recovered at 6 months

The Lightning process is used by children with CFS/ME in the UK but with no evidence of effectivness

What this study adds

At 6 months, children who received LP in addition to SMC had better physical function, fatigue and less anxiety

At 12 months, children who received LP in addition to SMC had better fatigue, anxiety, depression and school attendance.

Adding LP is probably cost effective but not all children wish to take part.

References

1. Chalder T, Goodman R, Wessely S, et al. Epidemiology of chronic fatigue syndrome and self reported myalgic encephalomyelitis in 5-15 year olds: cross sectional study. *BMJ* 2003;327(7416):654-~~55.~~5. doi: 10.1136/bmj.327.7416.654
2. Crawley EM, Emond AM, Sterne JA. Unidentified Chronic Fatigue Syndrome/myalgic encephalomyelitis (CFS/ME) is a major cause of school absence: surveillance outcomes from school-based clinics. *BMJ Open* 2011;1(2):e000252. doi: bmjopen-2011-000252 [pii];10.1136/bmjopen-2011-000252 [doi]
3. Crawley E, Hughes R, Northstone K, et al. Chronic ~~disabling fatigue~~Disabling Fatigue at ~~age~~Age 13 and ~~association with family adversity.~~Association With Family Adversity. *Pediatrics* 2012;130(1):~~e71-e79.~~E71-E79. doi: 10.1542/peds.2011-2587
4. Nijhof SL, Maijer K, Bleijenberg G, et al. Adolescent chronic fatigue syndrome: prevalence, incidence, and morbidity. *Pediatrics* 2011;127(5):e1169-e75. doi: peds.2010-1147 [pii];10.1542/peds.2010-1147 [doi]
5. Bould H, Collin SM, Lewis G, et al. Depression in paediatric chronic fatigue syndrome. *Arch Dis Child* 2013;98(6):425-8. doi: 10.1136/archdischild-2012-303396 [published Online First: 2013/04/27]
6. Fisher H, Crawley E. Why do young people with CFS/ME feel anxious? A qualitative study. *Clinical child psychology and psychiatry* 2013;18(4):556-73. doi: 10.1177/1359104512460862
7. Crawley E, Sterne JA. Association between school absence and physical function in paediatric chronic fatigue syndrome/myalgic encephalopathy. *ArchDisChild* 2009;94(10):752-56.
8. van Geelen SM, Bakker RJ, Kuis W, et al. Adolescent chronic fatigue syndrome: a follow-up study. *ArchPediatrAdolescMed* 2010;164(9):810-14.
9. Carter BD, Edwards JF, Kronenberger WG, et al. Case control study of chronic fatigue in pediatric patients. *Pediatrics* 1995;95(2):179-86.
10. Missen A, Hollingworth W, Eaton N, et al. The financial and psychological impacts on mothers of children with chronic fatigue syndrome (CFS/ME). *Child Care Health Dev* 2012;38(4):505-12. doi: 10.1111/j.1365-2214.2011.01298.x [published Online First: 2011/09/02]
11. Royal College of Paediatrics and Child Health. Evidence Based Guideline for the Management of CFS/ME (Chronic Fatigue Syndrome/Myalgic Encephalopathy) in Children and Young People. London, 2004.
12. NICE. Chronic fatigue syndrome/myalgic encephalomyelitis (or encephalopathy): Diagnosis and management of CFS/ME in adults and children (NICE guidelines CG53). London, 2007.
13. Rangel L, Garralda ME, Hall A, et al. Psychiatric adjustment in chronic fatigue syndrome of childhood and in juvenile idiopathic arthritis. *PsycholMed* 2003;33(2):289-97.
14. Rangel L, Garralda ME, Levin M, et al. The course of severe chronic fatigue syndrome in childhood. *JRSocMed* 2000;93(3):129-34.
15. Chambers D, Bagnall AM, Hempel S, et al. Interventions for the treatment, management and rehabilitation of patients with chronic fatigue syndrome/myalgic encephalomyelitis: an updated systematic review. ~~Journal of the Royal Society of Medicine~~*J R Soc Med* 2006;99(10):506-20.
16. Smith SN, Crawley E. Is there effective behavioural treatment for children with chronic fatigue syndrome/myalgic encephalomyelitis? *Arch Dis Child* 2013;98(7):561-3. doi: 10.1136/archdischild-2013-304307 [published Online First: 2013/06/13]
17. Stulemeijer M, de Jong LW, Fiselier TJ, et al. Cognitive behaviour therapy for adolescents with chronic fatigue syndrome: randomised controlled trial. *BMJ* 2005;330(7481):14.

Formatted: Font: Not Bold
Formatted: Font: Italic
Formatted: Font: Not Bold
Formatted: Font: Italic
Formatted: Font: Not Bold
Formatted: Font: Italic
Formatted: Font: Not Bold
Formatted: Font: Italic
Formatted: Font: Not Bold
Formatted: Font: Italic
Formatted: Font: Not Bold
Formatted: Font: Italic
Formatted: Font: Not Bold
Formatted: Font: Italic
Formatted: Font: Not Bold
Formatted: Font: Italic
Formatted: Font: Not Bold
Formatted: Font: Italic
Formatted: Font: Not Bold
Formatted: Font: Not Bold
Formatted: Font: Italic
Formatted: Font: Not Bold
Formatted: Font: Italic
Formatted: Font: Not Bold

18. Al-Haggar M S, Al-Naggar Z A, Abdel-Salam M A. Biofeedback and cognitive behavioural therapy for Egyptian adolescents suffering from ~~chronci~~chronic fatigue syndrome. . *J Paediatric Neurol* 2006;4:8.
19. Nijhof SL, Bleijenberg G, Uiterwaal CS, et al. Effectiveness of internet-based cognitive behavioural treatment for adolescents with chronic fatigue syndrome (FITNET): a randomised controlled trial. *Lancet* 2012;379(9824):1412-18.
20. Chalder T, Deary V, Husain K, et al. Family-focused cognitive behaviour therapy versus psycho-education for chronic fatigue syndrome in 11- to 18-year-olds: a randomized controlled treatment trial. *Psychol Med* 2010;40(8):1269-79.
21. Lloyd S, Chalder T, Rimes KA. Family-focused cognitive behaviour therapy versus psycho-education for adolescents with chronic fatigue syndrome: Long-term follow-up of an RCT. *BehavResTher* 2012;50(11):719-25. doi: S0005-7967(12)00132-5 [pii];10.1016/j.brat.2012.08.005 [doi]
22. Nijhof SL, Priesterbach LP, Uiterwaal CS, et al. Internet-based therapy for adolescents with chronic fatigue syndrome: long-term follow-up. *Pediatrics* 2013;131(6):e1788-95. doi: 10.1542/peds.2012-2007
23. Reme SE, Archer N, Chalder T. Experiences of young people who have undergone the Lightning Process to treat chronic fatigue syndrome/myalgic encephalomyelitis--a qualitative study. ~~British journal of health psychology~~*Br J Health Psychol* 2013;18(3):508-25. doi: 10.1111/j.2044-8287.2012.02093.x
24. Crawley E, Mills N, Beasant L, et al. The feasibility and acceptability of conducting a trial of specialist medical care and the Lightning Process in children with chronic fatigue syndrome: feasibility randomized controlled trial (SMILE study). *Trials* 2013;14~~.~~:415. doi: 10.1186/1745-6215-14-415
25. Crawley E, Mills N, Hollingworth W, et al. Comparing specialist medical care with specialist medical care plus the Lightning Process for chronic fatigue syndrome or myalgic encephalomyelitis (CFS/ME): study protocol for a randomised controlled trial (SMILE Trial). *Trials* 2013;14:444. doi: 10.1186/1745-6215-14-444 [published Online First: 2013/12/29]
26. Ware JE, Sherbourne CD. The MOS 36-item short-form health survey (SF-36). I. Conceptual framework and item selection. *MedCare* 1992;30(6):473-83.
27. ~~Chalder T, Berelowitz G, Pawlikowska T, et al. Development of a fatigue scale. JPsychosomRes 1993;**37**(2):147-53.~~Parslow R, Patel A, Beasant L, et al. What matters to children with CFS/ME? A conceptual model as the first stage in developing a PROM. *Arch Dis Child* 2015;100(12):1141-7. doi: 10.1136/archdischild-2015-308831 [published Online First: 2015/10/11]

~~28.~~ 28. Chalder T, Berelowitz G, Pawlikowska T, et al. Development of a fatigue scale. *J Psychosom Res* 1993;37(2):147-53.

~~Ravens-Sieberer U, Wille N, Badia X, et al. Feasibility, reliability, and validity of the EQ-5D-Y: results from a multinational study. *QualLife Res* 2010;19:887-97.~~

29. White D, Leach C, Sims R, et al. Validation of the Hospital Anxiety and Depression Scale for use with adolescents. *BrJPsychiatry* 1999;175:452-54.
30. Spence SH, Barrett PM, Turner CM. Psychometric properties of the Spence Children's Anxiety Scale with young adolescents. *JAnxietyDisord* 2003;17(6):605-25.
31. Ravens-Sieberer U, Wille N, Badia X, et al. Feasibility, reliability, and validity of the EQ-5D-Y: results from a multinational study. *QualLife Res* 2010;19:887-97.

32. Reilly MC, Zbrozek AS, Dukes EM. The validity and reproducibility of a work productivity and activity impairment instrument. ~~PharmacoEconomics~~*Pharmacoeconomics* 1993;4(5):353-65.

~~32~~33. Wyrwich KW, Tierney WM, Babu AN, et al. A comparison of clinically important differences in health-related quality of life for patients with chronic lung disease, asthma, or heart disease. *Health* ~~services research~~*Serv Res* 2005;40(2):577-91. doi: HESR373 [pii];10.1111/j.1475-6773.2005.00373.x [doi]

Formatted: Font: Italic
Formatted: Font: Not Bold
Formatted: Font: Italic
Formatted: Font: Not Bold
Formatted: Font: Italic
Formatted: Font: Not Bold
Formatted: Font: Italic
Formatted: Font: Not Bold
Formatted: Font: Italic
Formatted: Font: Not Bold
Formatted: Font: Not Bold
Formatted: Font: Italic
Formatted: Font: Not Bold
Formatted: Font: Italic
Formatted: Font: Not Bold
Formatted: Font: Italic
Formatted: Font: Not Bold
Formatted: Font: Italic
Formatted: Font: Not Bold
Formatted: Font: Italic
Formatted: Font: Not Bold
Formatted: Font: Italic
Formatted: Font: Not Bold
Formatted: Font: Italic
Formatted: Font: Not Bold
Formatted: Font: Not Bold
Formatted: Font: Italic
Formatted: Font: Not Bold

~~33~~34. NICE. Guide to the methods of technology appraisal 2013 04 April 2013.
~~34~~35. Department of H. Reference costs 2012 to 2013. *NHS reference costs* 2013 21 November 2013.
~~35~~36. Curtis L. Unit Costs of Health and Social Care. 2013.
~~36~~37. Prescribing and Primary Care Team HasCIC. Prescription Cost Analysis - England, 2012 [NS]. 2013 04 April 2013.
~~37~~38. Hill A, Cooper M, Pybis J, et al. Evaluation of the Welsh School-based Counselling Strategy: Final Report. , . In: Research WGS, ed., 2011.
~~38~~39. Dolan P. Modeling valuations for EuroQol health states. ~~Medical care~~*Med Care* 1997;35(11):1095-108.
~~39~~40. Manca A, Hawkins N, Sculpher MJ. Estimating mean QALYs in trial-based cost-effectiveness analysis: the importance of controlling for baseline utility. *Health* ~~economics~~*Econ* 2005;14(5):487-96. doi: 10.1002/hec.944 [published Online First: 2004/10/22]
~~40~~41. White P, Goldsmith K, Johnson A, et al. Comparison of adaptive pacing therapy, cognitive behaviour therapy, graded exercise therapy, and specialist medical care for chronic fatigue syndrome (PACE): a randomised trial. *Lancet* 2011;377(9768):823-36.
~~41. Nursing RCo. Pay rates 2012/13. Agenda for Change pay bands effective from 1 April 2012. Secondary Pay rates 2012/13. Agenda for Change pay bands effective from 1 April 2012 2012.~~

42. Brigden A, Parslow RM, Gaunt D, et al. Defining the minimally clinically important difference of the SF-36 physical function subscale for paediatric CFS/ME: triangulation using three different methods. *Health and quality of life outcomes* 2018;16(1):202. doi: 10.1186/s12955-018-1028-2 [published Online First: 2018/10/21]

Formatted: Font: Italic
Formatted: Font: Not Bold
Formatted: Font: Italic
Formatted: Font: Not Bold
Formatted: Font: Italic
Formatted: Font: Not Bold
